# Sulfonamide as amide isostere for fine-tuning the gelation properties of physical gels†

**DOI:** 10.1039/d0ra00943a

**Published:** 2020-03-20

**Authors:** Juan V. Alegre-Requena, Santiago Grijalvo, Diego Sampedro, Judith Mayr, César Saldías, José Juan Marrero-Tellado, Ramón Eritja, Raquel P. Herrera, David Díaz Díaz

**Affiliations:** Institut für Organische Chemie, Universität Regensburg Universitätsstr. 31 93053 Regensburg Germany David.Diaz@chemie.uni-regensburg.de; Laboratorio de Organocatálisis Asimétrica, Departamento de Química Orgánica, Instituto de Síntesis Química y Catálisis Homogénea (ISQCH), CSIC-Universidad de Zaragoza Pedro Cerbuna 12 50009 Zaragoza Spain raquelph@unizar.es; Biomedical Research Networking Centre in Bioengineering, Biomaterials and Nanomedicine (CIBER-BBN) Jordi Girona 18-26 08034 Barcelona Spain; Institute of Advanced Chemistry of Catalonia (IQAC-CSIC) Jordi Girona 18-26 08034 Barcelona Spain; Departamento de Química, Universidad de La Rioja Madre de Dios, 51 26006 Logroño Spain; Departamento de Química Física, Facultad de Química y de Farmacia, Pontificia Universidad Católica de Chile Macul 7820436 Santiago Chile; Departamento de Química Orgánica, Universidad de La Laguna Avda. Astrofísico Francisco Sánchez 38206 La Laguna Tenerife Spain ddiazdiaz@ull.edu.es; Instituto Universitario de Bio-Orgánica Antonio González, Universidad de La Laguna Avda. Astrofísico Francisco Sánchez 2 38206 La Laguna Tenerife Spain

## Abstract

(*S*)-2-Stearamidopentanedioic acid (C_18_-Glu) is a known LMW gelator that forms supramolecular gels in a variety of solvents. In this work, we have carried out the isosteric substitution of the amide group by a sulfonamide moiety yielding the new isosteric gelator (*S*)-2-(octadecylsulfonamido)pentanedioic acid (Sulfo-Glu). The gelation ability and the key properties of the corresponding gels were compared in terms of gelation concentration, gel-to-sol transition temperature, mechanical properties, morphology, and gelation kinetics in several organic solvents and water. This comparison was also extended to (*S*)-2-(4-hexadecyl-1*H*-1,2,3-triazol-4-yl)pentanedioic acid (Click-Glu), which also constitutes an isostere of C_18_-Glu. The stabilizing interactions were explored through computational calculations. In general, Sulfo-Glu enabled the formation of non-toxic gels at lower concentrations, faster, and with higher thermal-mechanical stabilities than those obtained with the other isosteres in most solvents. Furthermore, the amide-sulfonamide isosteric substitution also influenced the morphology of the gel networks as well as the release rate of an embedded antibiotic (vancomycin) leading to antibacterial activity *in vitro* against *Staphylococcus aureus*.

## Abstract 2

Amide–sulfonamide isosteric substitution in a low-molecular-weight gelator enables fine-tuning of its gelation ability and the properties of the corresponding supramolecular gels.

## Graphical abstract

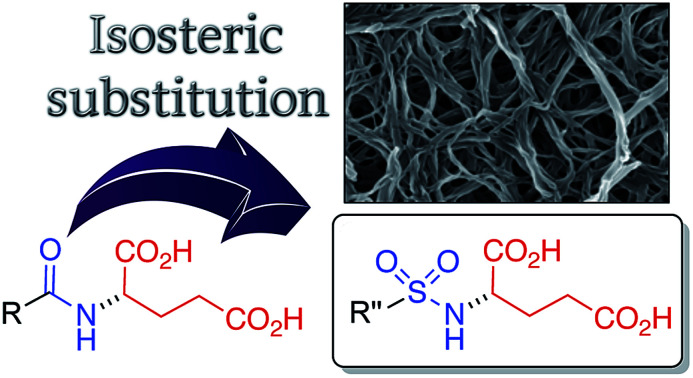

## Introduction

Gel materials have gained increasing attention during the last few decades^1–3^ due to their interesting molecular architectures and potential for high-tech applications in various fields,^4,5^ such as the preparation of conductive scaffolds,^6,7^ sensors,^8^ liquid crystals,^9^ templates for cell growth^10^ and inorganic structures,^11^ cosmetics,^12^ catalysis,^13,14^ and food industries.^15^ In contrast to chemical gels^16^ made of covalent bonds, physical (or supramolecular) gels^17–19^ are formed by self-assembly of low-molecular-weight (LMW) molecules (gelators) in solution into 1D nanofibers followed by their entanglement forming a 3D scaffold. The solid-like appearance of the gels derives from the immobilization of solvent molecules into the interstices of the 3D network due to surface tension and capillary forces. Remarkably, this phenomenon can increase the viscosity of the medium by factors of up to 10^10^ depending on the chemical nature of the solvent and gelator. Due to the non-covalent nature of the molecular interactions (*e.g.* hydrogen-bonding, π-stacking, van der Waals) that drive the self-assembly process, most supramolecular gels show reversible gel-to-sol phase transitions when exposed to external stimuli (*e.g.* temperature, pH, ionic strength).^20–22^ Notably, the use of supramolecular hydrogels have been recently reported in many biomedical applications like wound healing,^23^ being also employed as drug delivery systems for the release of anticancer^24^ and antibacterial^25–28^ drugs, among others.

**Fig. 1:**
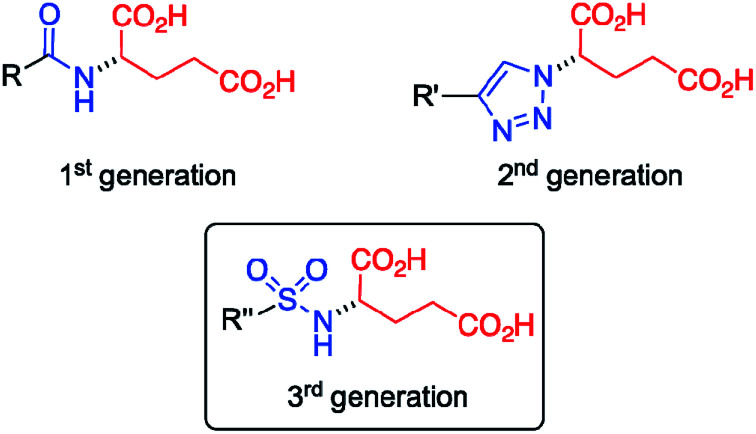
Different generations of LMW isosteric gelators derived from l-glutamic acid referred in this work. Isosteric core: amide (1^st^ generation); 1,2,3-triazole (2^nd^ generation); sulfonamide (3^rd^ generation). *R*, *R*′ and *R*″ = lineal aliphatic chains.

Our group has demonstrated that the isosteric substitution^29^ of amides by 1,2,3-triazole groups in LMW gelators enables the adjustment of gel properties.^30–32^ This approach have been successfully applied to different gel materials.^33,34^ In general, isosteres have similar molecular shape, volume, electronic distribution, and exhibit comparable physical properties.^35,36^ In a previous work,^24^ we replaced the amide group of an amphiphilic LMW gelator based on glutamic acid (Fig. 1, 1^st^ generation) by a 1,2,3-triazole ring to afford the corresponding click-isostere (Fig. 1, 2^nd^ generation), which provided gels with enhanced properties in several solvents. In this study, we wondered if other amide isosteres could also be used for tuning the gel properties. Thus, we focused our attention on the sulfonamide group, a functional moiety structurally more similar to amides compared to 1,2,3-triazoles, which preserves the H-bond donor and O acceptor sites of the amide group (Fig. 1, 3^rd^ generation).

## Experimental

### Materials

Unless otherwise specified, all reagents, starting materials and solvents (p.a. grade) were purchased from commercial suppliers and used as received without further purification. Double-distilled water was purified additionally using a Millipore water-purifying system (Merck) prior usage. Xylene as mixture of isomers was used after double-distillation. Isosteric gelators (*S*)-2-(4-hexadecyl-1*H*-1,2,3-triazol-4-yl)pentanedioic acid (Click-Glu) and (*S*)-2-stearamidopentanedioic acid (C_18_-Glu) were prepared as previously described.^24^

### Characterization methods

#### Characterization of compounds

^1^H NMR spectra and ^13^C APT-NMR spectra were recorded at 300 MHz and 75 MHz respectively using a Bruker ARX 300 MHz spectrometer. CDCl_3_ and DMSO-*d*_6_ were used as deuterated solvents. Chemical shifts were reported in the *δ* scale relative to residual CHCl_3_ (7.26 ppm) and DMSO (2.50 ppm) for ^1^H NMR and to the central line of CDCl_3_ (77.16 ppm) and DMSO-*d*_6_ (39.52 ppm) for ^13^C APT-NMR. Coupling constants, *J*, are given in Hertz. Coupling patterns are indicated using the following abbreviations: s = singlet, br s = broad singlet, d = doublet, t = triplet, m = multiplet. The estimated error of the reported values is 0.01 ppm (*δ*, ^1^H NMR), 0.1 ppm (*δ*, ^13^C NMR) and 0.1 Hz (*J*, coupling constant). NMR spectra are provided in the ESI.†

High-resolution mass spectroscopy (HRMS) measurements were carried out by ESI ionization method on a mass analyzer type MicroTof-Q.

Fourier transform infrared (FT-IR) spectra were recorded at room temperature using an Excalibur FTS 3000 FT-IR spectrometer (Biorad) equipped with a single reflection ATR (attenuated total reflection) accessory (Golden Gate, Diamond).

Thin layer chromatography (TLC) analyses were performed using fluorescent-indicating plates (aluminum sheets precoated with silica gel 60 F_254_, thickness 0.2 mm, Merck), and visualization achieved by UV light (*λ*_max_ = 254 nm) and staining with phosphomolybdic acid.

Melting points were measured on a GallenKamp MPD 350 BM 2.5 apparatus.

Specific rotation calculations were made in chloroform or DMSO using a Jasco P-1020 polarimeter.

#### Characterization of gel materials

Critical gelation concentration (CGC) values (defined as the minimum concentration of compound where gelation is observed) were estimated by continuously adding aliquots of solvent (0.05–0.1 mL) into vials containing 20 mg of Sulfo-Glu and performing a typical heating-cooling protocol for gel formation until no gelation was observed. The starting point for CGC determinations was 200 g L^−1^.

Thermal gel-to-sol transition temperature (*T*_gel_) values were determined using a custom made set-up in which the sealed vial containing the gel was placed into a mold of an alumina block and heated up at approximately 5 °C min^−1^ using an electric heating plate equipped with a temperature control couple.^37^ The values obtained with this equipment were previously verified by DSC measurements.^31^ Herein, the temperature at which the gel started to break was defined as *T*_gel_. Each measurement was made at least by duplicate and the average value reported. *T*_gel_ values were found almost unaltered within a difference of 1–2 °C after several heating–cooling cycles.

Oscillatory rheology was performed with an AR 2000 Advanced rheometer (TA Instruments) equipped with a Julabo C cooling system. A 1000 µm gap setting and a torque of 5 × 10^−4^ N m^−1^ at 25 °C were used for the measurements in a plain-plate (20 mm, stainless steel). The following experiments were carried out for each sample: (a) Dynamic Strain Sweep (DSS): variation of *G*′ (storage modulus) and *G*″ (loss modulus) with strain (from 0.01 to 100%); (b) Dynamic Frequency Sweep (DFS): variation of *G*′ and *G*″ with frequency (from 0.1 to 10 Hz at 0.1% strain); (c) Dynamic Time Sweep (DTS): variation of *G*′ and *G*″ with time keeping the strain and frequency values constant and within the linear viscoelastic regime as determined by DSS and DFS measurements (strain = 0.1% strain; frequency = 1 Hz).

Differential scanning calorimetry (DSC) measurements were performed on a Mettler Toledo Differential Scanning Calorimeter using a DSC 30 measuring cell. The DSC thermogram was obtained under dynamic argon atmosphere (gas flow rate = 25 mL min^−1^) at a heating rate of 5 °C min^−1^. Samples were placed in closed aluminum pans (Mettler Toledo). An empty sample holder was used as reference and the runs were performed by heating the samples from 20 to 60 °C (EtOAc gel). The values were reported as the average of two independent measurements.

FT-IR spectra of gel-based materials were recorded as indicated above for the characterization of compounds.

Morphological characterization of the bulk samples was carried out by Field Emission Scanning Electron Microscopy (FESEM) and Transmission Electron Microscopy (TEM). (a) FESEM: images were obtained with a Zeiss Merlin, Field Emission Scanning Electron Microscope (0.8 nm resolution) equipped with a digital camera (SAI, Universidad de Zaragoza) and operating at 3 kV and 158 pA. Sample preparation: xerogel specimens were prepared by freeze-drying. Prior to imaging, so obtained fibrous solid was placed on carbon tape and shielded by Pt (40 mA during 30 s; film thickness ≈ 5 nm). (b) TEM: images were recorded using a JEOL-2000 FXII transmission electron microscope (0.28 nm resolution) equipped with a CCD Gatan 694 digital camera (SAI, Universidad de Zaragoza) and operating at 200 kV (accelerating voltage). Sample preparation: 10 µL of the gel suspension was allowed to adsorb onto carbon-coated grids (300 mesh, from Aname). The grid was placed over a piece of filter paper in order to absorb the excess of solvent.

### Synthesis of compounds

**Fig. 2:**
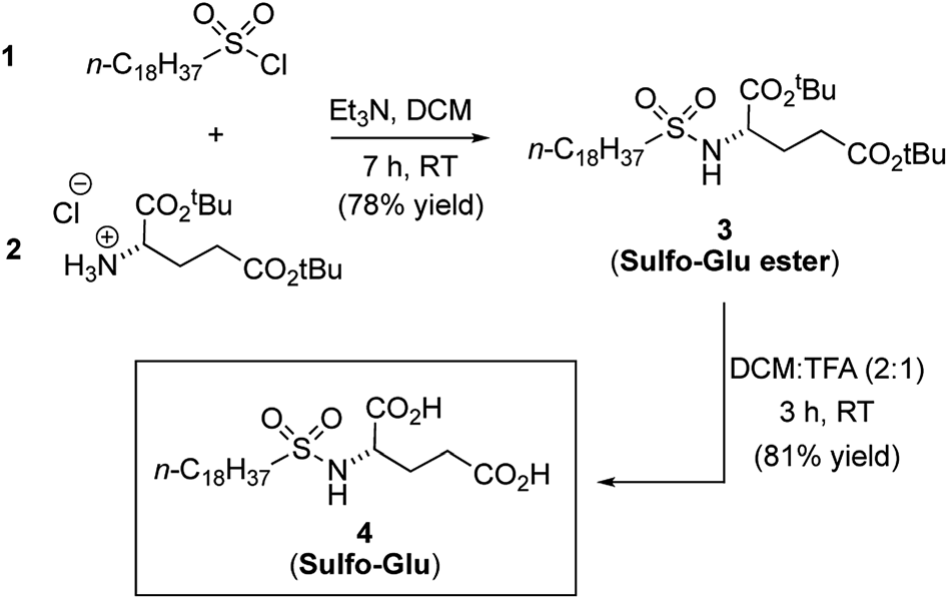
Synthetic scheme for the preparation of gelator 4 (Sulfo-Glu).

See Fig. 2 for synthetic scheme and compounds numeration.

#### (*S*)-Di-*tert*-butyl 2-(octadecylsulfonamido)pentanedioate (3, Sulfo-Glu ester)

To a mixture of di-*tert*-butyl-l-glutamate hydrochloride salt (2) (4 mmol, 1.21 g) in dry DCM (30 mL), Et_3_N (12 mmol, 1.7 mL) was added at RT. Then, a solution of octadecane-1-sulfonyl chloride (1) (4 mmol, 1.49 g) in dry DCM (10 mL) was added dropwise. Adduct 3 was isolated by flash chromatography (SiO_2_, from hexane–EtOAc 95 : 5 to hexane–EtOAc 8 : 2) after 17 h of reaction at RT as a white solid in 78% yield (1.8 g). Mp 54–57 °C. [*α*]^D^_22_ = +2.3 (*c* = 0.81, CHCl_3_).^1^H NMR (300 MHz, CDCl_3_) *δ* 4.90 (d, *J* = 9.1 Hz, 1H, SO_2_NH), 3.98 (m, 1H, SO_2_NH–CH̲), 3.03–2.86 (m, 2H, CH̲_2_–SO_2_NH), 2.44–2.33 (m, 2H, Aliph-CH_2_), 2.16–2.02 (m, 1H, Aliph-CH_2_), 1.93–1.15 (m, 51H, Aliph-CH_2_ and C–(CH_3_)_3_), 0.94–0.81 (m, 3H, Aliph-CH_3_). ^13^C NMR (75 MHz, CDCl_3_) *δ* 172.1 (s, 1C, C=O), 171.4 (s, 1C, C=O), 83.1 (s, 1C, C̲–(CH_3_)_3_), 81.0 (s, 1C, C̲–(CH_3_)_3_), 56.9–55.1 (different conformations, 1C, SO_2_NH–CH), 53.7 (s, 1C, CH_2_–SO_2_NH), 32.1 (s, 1C, Aliph-CH_2_), 31.3 (s, 1C, Aliph-CH_2_), 29.8 (s, 4C, Aliph-CH_2_), 29.7 (s, 1C, Aliph-CH_2_), 29.7 (s, 1C, Aliph-CH_2_), 29.5 (s, 1C, Aliph-CH_2_), 29.4 (s, 1C, Aliph-CH_2_), 29.2 (s, 1C, Aliph-CH_2_), 28.9 (s, 1C, Aliph-CH_2_), 28.8 (s, 1C, Aliph-CH_2_), 28.5 (s, 1C, Aliph-CH_2_), 28.3 (s, 2C, C–(C̲H_3_)_3_), 28.2 (s, 2C, C–(C̲H_3_)_3_), 28.1 (s, 1C, C–(C̲H_3_)_3_), 28.1 (s, 1C, C–(C̲H_3_)_3_), 27.5 (s, 1C, Aliph-CH_2_), 27.4 (s, 1C, Aliph-CH_2_), 23.6 (s, 1C, Aliph-CH_2_), 22.8 (s, 1C, Aliph-CH_2_). FT-IR (KBr film) (cm^−1^) *ν* 3280, 2924, 2854, 1732, 1458, 1393, 1368, 1337, 1256, 1150, 981, 914, 845, 720. HRMS (ESI+) calcd C_31_H_61_NNaO_6_S 598.4112; found 598.4125 [M + Na].

#### (*S*)-2-(Octadecylsulfonamido)pentanedioic acid (4, Sulfo-Glu)

Compound 3 (3.12 mmol, 1.8 g) was dissolved in 20 mL of a mixture DCM : TFA (2 : 1) at RT. After 3 h of reaction, the solvents were evaporated and the crude was suspended in DCM, filtrated and dried under high vacuum. Compound 4 was obtained as a white solid in 81% yield (1.2 g). Mp 121–125 °C. [*α*]^D^_22_ = −11.8 (*c* = 0.51, DMSO). ^1^H NMR (300 MHz, DMSO-*d*_6_) *δ* 12.49 (br s, 2H, CO_2_H), 7.53 (d, *J* = 9.1 Hz, 1H, SO_2_NH), 3.84 (m, 1H, SO_2_NH–CH̲), 3.02–2.86 (m, 2H, CH̲_2_–SO_2_NH), 2.42–2.23 (m, 2H, Aliph-CH_2_), 2.02–1.87 (m, 1H, Aliph-CH_2_), 1.80–0.96 (m, 33H, Aliph-CH_2_), 0.92–0.79 (m, 3H, Aliph-CH_3_). ^13^C NMR (75 MHz, DMSO-*d*_6_) *δ* 173.7 (s, 1C, C=O), 173.4–173.3 (different conformations, 1C, C=O), 55.3–54.0 (different conformations, 1C, SO_2_NH–CH), 52.5 (s, 1C, CH_2_–SO_2_NH), 31.3 (s, 1C, Aliph-CH_2_), 29.8 (s, 1C, Aliph-CH_2_), 29.0 (s, 10C, Aliph-CH_2_), 28.8 (s, 1C, Aliph-CH_2_), 28.7 (s, 1C, Aliph-CH_2_), 28.6 (s, 1C, Aliph-CH_2_), 27.6 (s, 1C, Aliph-CH_2_), 23.2 (s, 1C, Aliph-CH_2_), 22.1 (s, 1C, Aliph-CH_2_), 14.5 (1C, Aliph-CH_3_). FT-IR (solid) (cm^−1^) *ν* 3295, 2922, 2847, 1744, 1677, 1465, 1409, 1383, 1327, 1275, 1215, 1133, 1003, 906, 857, 794, 723. HRMS (ESI+) calcd C_23_H_45_NNaO_6_S 486.2860; found 486.2853 [M + Na].

### General procedure for the preparation of gels

Typically, Sulfo-Glu (20 mg) and increasing amounts of the desired solvent (the maximum concentration of these studies was 200 g L^−1^) were placed into a screw-capped glass vial (4 cm length × 1 cm diameter). Then, these vials were gently heated with a heat gun until the solid material was completely dissolved. Unless otherwise noted, the resulting isotropic solution was then spontaneously cooled down to RT. No control over temperature rate during the heating–cooling process was applied. The material was preliminarily classified as “gel” if it did not exhibit gravitational flow upon turning the vial upside-down at RT. The state was further confirmed by rheological measurements.

### Concentration dependence of gelation time and *T*_gel_

Samples containing a weighted amount of Sulfo-Glu and Milli-Q water (0.5 mL) were prepared in screw-capped vials at different concentrations ranging from 38 to 200 g L^−1^ (*i.e.* from GCG to a maximum where water could be absorbed). The samples were heated until turbid solutions were formed. Gelation times were determined upon cooling the samples to RT. After resting for 19 h, the *T*_gel_ values were measured as explained above.

### Stimuli response studies

Typically, samples containing Sulfo-Glu (60 mg, 0.13 µmol) and Milli-Q water (0.5 mL) were prepared in screw-capped glass vials (4 cm length × 1 cm diameter). The samples were tempered at 100 °C for 1 h and turbid solutions were formed. The heating was turned off and the samples were cooled to RT. After 19 h, stable gels were obtained, which were overlaid with different external additives (0.5 mL from a 0.1 M aqueous solution). The samples were stored at RT and monitored during 24 h.

### Cell viability studies

Cells metabolic activity was evaluated after 24 h of incubation at 37 °C using the MTT assay (MTT = 3-(4,5-dimethylthiazol-2-yl)-2,5-diphenyltetrazolium bromide). HeLa human tumor cells were exponentially passaged before they reached confluence. Cells were seeded into 96-well plates at a density of 6000 cells per well in 200 µL Dubelcco's Modified Eagle Medium (DMEM) supplemented with 10% fetal bovine serum (FBS). When cells reached 60–70% confluence, the growth media was replaced by fresh DMEM (198 µL). Isosteric gelators were incubated in the presence of HeLa cells at several concentrations (total volume 200 µL) for 8 h at 37 °C. Then, medium was discharged and replaced with fresh growth media. HeLa cells were incubated for 10 h. MTT solution (25 µL from 5 mg mL^−1^ stock solution in PBS) was added to each well and incubated again for 3 h. Finally, medium was changed and DMSO was added (150 µL) to dissolve the formazan crystals. All studies were made in six biological replicates. Absorbance was measured at 560 nm in a Glomax Multidetection System instrument (Promega). Results were expressed as a relative percentage to untreated control cells. Statistical differences between groups at significance levels >95% were calculated by Student's *t* test. In all cases, *p* values < 0.05 were regarded as significant. Numerical data are presented as mean ± standard deviation (SD).

### *In vitro* antimicrobial studies

#### Preparation of agar plates

A solution of 1% peptone, 0.5% yeast, 0.2% glucose and 1.5% of agar–agar (w/v) in Milli-Q water was autoclaved at 120 °C for 20 min and then shaken. After cooling the mixture to 95 °C, a Petri dish (8.5 cm diameter) was filled to half of the volume (*ca.* 25 mL). The dishes were shaken circularly on the bench to bring air bubbles to the edge. After cooling to RT, the plates were placed upside down to avoid condensed water to drop on the medium and stored in the refrigerator at 2–8 °C. *S. aureus* was seeded out evenly with a cotton bud on agar medium.

#### Preparation of antibiotic-containing hydrogel

Sulfo-Glu (60 mg, 0.13 µmol) and an aqueous solution of vancomycin (1 mL, *c* = 2 mg mL^−1^) were placed into a screw-capped vial. The mixture was heated until a homogeneous, turbid solution was formed. The hot mixture was transferred to a 2 mL syringe with cut cap and rested over night to obtain the corresponding stable hydrogels. In the same manner hydrogels without vancomycin but with pure water were prepared.

#### Antimicrobial tests with hydrogel pieces

Nearly round pieces of gels with and without vancomycin (*ca.* 9 mm diameter; 0.25 mL) were placed on the bacteria plates. The plates were stored at 37 °C for 24 h. The diameters of the clear zones surrounding the wells were measured. The antibiotic activity over time was estimated by subtracting the diameter of the central well for each sample.^38^

#### Antimicrobial tests with drug release supernatants

Filter papers were placed on the bacteria plates. After 24 h of drug release, the filters were soaked separately with 20 µL of (a) supernatant obtained from the drug release experiment, (b) pure phosphate buffer solution (PBS) and (c) aqueous vancomycin solution (*c* = 2 mg mL^−1^). The plates were stored at 37 °C for 24 h. The diameters of the clear zones surrounding the wells were measured.

#### Statistical analysis

All experiments were done in triplicate. Blank gels were always prepared and tested along with their drug-loaded counterparts. Data are presented as mean of three replicates for each experiment ± SD. Unpaired student-*t* test was used for comparing between two variables and probability values *P* value < 0.05 was considered significant.

### Typical procedure for drug release studies

Vancomycin-containing gels were stabilized for 21 h before measurements. After this time, the gels were overlaid with PBS buffer pH 7.4 (1 mL). Aliquots of 100 µL were removed at defined intervals from the release medium and diluted with fresh PBS buffer to 1 mL. The release medium was refilled with fresh PBS buffer (100 µL) to maintain infinite sink conditions. The concentration of vancomycin in the aliquots was measured by UV in a Varian Cary 50 UV-Vis Spectrophotometer using quartz cuvettes (path length, 0.5 cm) after proper calibration using the maximum absorbance of vancomycin in aqueous media at 280 nm. Samples were centrifuged (EBA 12 Hettich Zentrifugen) at 4000 rpm for 5 min before measurements. It was verified that degraded gel materials exhibited a minimum absorbance in the region of drug detection.

### Computational studies

All calculations were performed using the M06-L functional^39^ and the 6-31+G(d,p) basis set^40^ in the Gaussian 16 computer package.^41^ The M06-L functional was chosen due to its ability to describe non-bonding interactions for a wide range of compounds, especially for large systems. The M06-L functional has been shown to describe the geometry and interaction between complexes stabilized by non-covalent interactions, such as π–π stacking, with high accuracy.^42^ All geometry optimizations were performed in different implicit solvents to allow comparison with the experimental results. Water (*ε* = 78.3553), ethanol (*ε* = 24.852) and chloroform (*ε* = 4.7113) were chosen and described through a simple Self Consistent Reaction Field (SCRF) method. More specifically, the Polarizable Continuum Model^43,44^ (PCM) was used to represent bulk solvent effects. The stationary points and the zero-point energies and thermal corrections to the energy were computed through frequency calculations. Binding energies were corrected with the basis set superposition error (BSSE) by means of the standard counterpoise (CP) method.^45^

## Results and discussion

### Synthesis of gelator Sulfo-Glu

Sulfo-Glu was easily synthesized in two steps with *ca.* 63% overall yield. First, di-*tert*-butyl-l-glutamate hydrochloride salt (2) was reacted at RT with octadecane-1-sulfonyl chloride (1) in the presence of Et_3_N as neutralization base (Fig. 2). This step afforded the Sulfo-Glu ester intermediate in good yield (78%), which was subsequently treated with TFA to cleave the *tert*-butyl esters and form the desired Sulfo-Glu (81%).

### Solubility properties and gelation ability

The gelation capacity of Sulfo-Glu was studied in a variety of solvents of different nature (*i.e.* non-polar, polar protic and aprotic solvents) by applying a heating–cooling cycle as described in the experimental section (Table S1†). Materials that did not show gravitational flow when the vial was turned upside-down were preliminarily classified as stable gels. The viscoelastic nature of representative gels was later confirmed by oscillatory rheological experiments (see below). Stable gels were obtained in aromatic solvents (*e.g.* xylene, benzene, toluene, benzonitrile, chlorobenzene), chlorinated solvents (*e.g.* methylene chloride, chloroform), ethyl acetate, acetonitrile, glycerol and water. In contrast, only partial gels could be obtained at concentrations ≤200 g L^−1^ in ethers, acetone, hexane and monoalcohols. Interestingly, Sulfo-Glu enabled the formation of gels in several solvents that could not be gelled using the previous analogues 1,2,3-triazole (Click-Glu) and amide (C_18_-Glu) isosteric gelators^24^ (*e.g.* ethyl acetate, benzonitrile, chlorobenzene) (Table S2†). However, other solvents such as protic solvents (*e.g.* methanol, ethanol, propan-2-ol) and DMSO were only gelled by the 1,2,3-triazole and/or amide isosteres, while Sulfo-Glu afforded partial gels or clear solutions, respectively (Table S1†). Therefore, the isosteric replacement of the amide group in the parent LMW gelator by sulfonamide and 1,2,3-triazole groups clearly affects the intermolecular non-covalent interactions responsible for gel formation (*e.g.* gelator–gelator, gelator–solvent, aggregate–solvent interactions). Thus, this strategy allows expanding the pool of gels that can be formed on-demand using isosteric gelators.

**Fig. 3:**
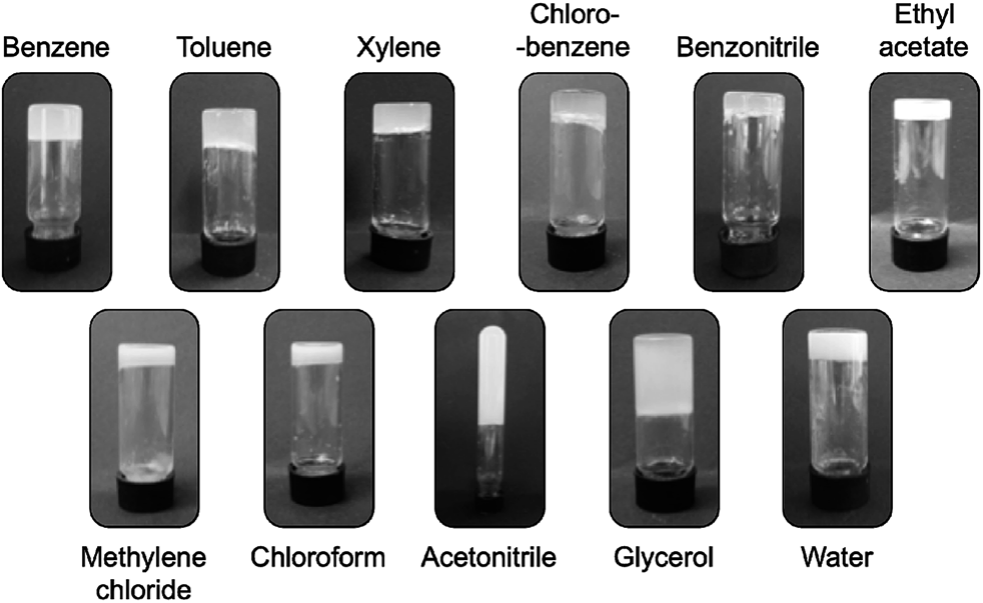
Digital photographs of selected gels made of Sulfo-Glu at the corresponding CGC in various organic solvents and water.

Fig. 3 shows photographs of upside-down vials containing gels made of Sulfo-Glu at the corresponding critical gelation concentration (CGC). In general, most gels were opaque -albeit in different degree depending on the solvent-suggesting the formation of aggregates larger than the wavelength of visible light (*ca.* 350–750 nm), which was in good agreement with the observations made by electron microscopy (see below). The optical appearance of the gels was very similar regardless the isosteric gelator used for their preparation.^24^

### Gel-to-sol transition temperature (*T*_gel_), gelation time and critical gelation concentration (CGC)

**Fig. 4:**
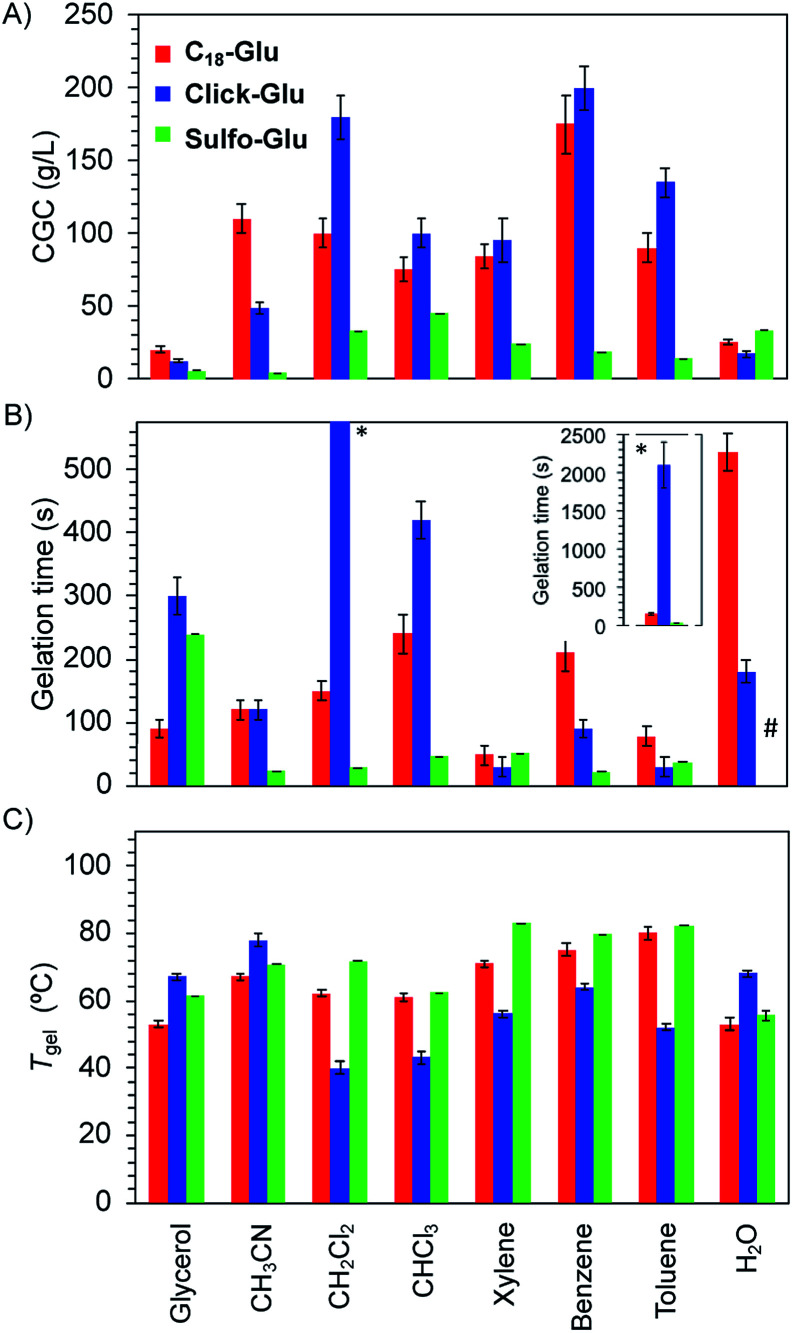
Compararison of (A) CGC, (B) gelation time, and (C) *T*_gel_ values for the gels made of Sulfo-Glu, Click-Glu and C_18_-Glu in 8 solvents. Both *T*_gel_ and gelation time values were measured at the highest CGC of the three isosteres. Each experiment was repeated at least three separate times and the error bars show the SD. ^#^ For the sake of clarity, the gelation time in water for Sulfo-Glu (>12 h) was omitted from the plot.

Significant differences were found regarding *T*_gel_, gelation time and CGC values for gels made of each isostere in 8 solvents (Fig. 4). Thus, isosteric substitution in LMW gelators greatly impacts their self-assembly properties. In general, gels showing high thermal resistance (high *T*_gel_), fast gelation times and low CGC are promising candidates for practical applications. As expected for supramolecular gels, *T*_gel_ values gradually increased with the increasing gelator concentration regardless the gelled solvent due to the formation of denser packed supramolecular networks (Fig. S1†). Similarly, an exponential decrease of the gelation time took place as the concentration increased (Fig. S2†). This behavior was observed with all the isosteric gelators.^24^

CGC values for the gels made of Sulfo-Glu were always lower in all organic solvents than those obtained with Click-Glu and C_18_-Glu isosteres (Fig. 4A). Remarkably, in some solvents such as acetonitrile, toluene and benzene, the CGC was found to be up to 32 times lower in the case of Sulfo-Glu. In contrast, the CGC determined in water was slightly higher in the case of Sulfo-Glu. This may be related to the different stabilizing interactions found for each compound (see below). For instance, water may interfere with the hydrogen bonding between dimers of Sulfo-Glu. As this was the main interaction found for this compound, a decrease in the stabilization of dimers in water could be related to the higher CGC value.

Furthermore, Sulfo-Glu showed from 5 to 75 times faster gelation in halogenated solvents such as CH_2_Cl_2_ and CHCl_3_, as well as in CH_3_CN, compared to Click-Glu and C_18_-Glu isosteres (Fig. 4B). In aromatic solvents, the three gelators showed similar gelation kinetics, except in the case of benzene, in which Sulfo-Glu gelled the solvent 9 times faster. In protic organic solvents, such as glycerol, Sulfo-Glu and Click-Glu showed comparable results, while C_18_-Glu formed gels *ca.* 2.5 faster than Sulfo-Glu. Again, in the case of water Sulfo-Glu showed poorer results compared to the other isosteres (*i.e.*C_18_-Glu and Click-Glu formed hydrogels within minutes while hydrogelation by Sulfo-Glu took place after 12 h).

All Sulfo-Glu gels exhibited *T*_gel_ values in the range of *ca.* 61–85 °C. Interestingly, the *T*_gel_ of the Sulfo-Glu gel made in CH_2_Cl_2_ was much higher than the boiling point of the solvent (*i.e.* bp = 39.6 °C; *T*_gel_ = 69 °C). Comparative measurements showed that Sulfo-Glu gels are thermally more stable than the analogue gels made of C_18_-Glu in many solvents (*i.e.*Sulfo-Glu gels displayed up to 12 °C higher *T*_gel_ values than C_18_-Glu gels). Sulfo-Glu gels also showed significantly higher *T*_gel_ values than Click-Glu gels in aromatic and halogenated solvents (Δ*T*_gel_ ∼ 15–31 °C). The exception was found for the gels made in acetonitrile and in water, where Click-Glu gels showed *ca.* 8–13 °C higher *T*_gel_ than Sulfo-Glu gels (Fig. 4C). In these solvents (acetonitrile, water) Sulfo-Glu and C_18_-Glu gels showed comparable *T*_gel_ values.

It should be emphasized that the measured *T*_gel_ values for each compound was found to be loosely related to the thermodynamic properties, as reflected by the calculated stabilization energy (*vide infra*). This qualitative trend links the macroscopic gelation properties with the computed stabilization energy for the series of isosteres for each solvent, and the different solvents (water, ethanol and chloroform for the computed data).

### Stability and stimuli-responsive properties

Gels prepared with the three isosteres were thermo-reversible, as expected for supramolecular gels, and stable for several months at RT without detriment of their consistency and visual appearance. In general, the response of Sulfo-Glu hydrogels towards external stimuli was similar to that of Click-Glu and C_18_-Glu gels in the presence of acidic, neutral and alkaline solutions, as well as in the presence of different metal salts (Fig. S3†). Specifically, an irreversible gel-to-sol transition took place in the presence of basic aqueous solutions such as aqueous NaOH or carbonate buffer (pH 9.3), whereas these hydrogels remained stable under neutral (H_2_O, phosphate buffer pH 7.0) or acidic (HCl, acetate buffer pH 5.0) conditions. Furthermore, the addition of salt solutions (*i.e.* AgNO_3_, Cu(NO_3_)_2_, CuSO_4_, Fe(NO_3_)_3_, Bu_4_NF) did not visually affect the stability of the gel, albeit the original white gels turned colored (grayish, bluish, orangish), most likely due to the incorporation of the metal ions into the gel networks (Fig. S3†). The major differences between the hydrogels made of the three isosteres were found upon addition of organic solvents. While Click-Glu and C_18_-Glu formed hydrogels that were stable in contact with many organic solvents, Sulfo-Glu hydrogels were partially or totally destroyed after addition of diverse organic solvents such as hexane, methylene chloride, ethyl acetate, and methanol – the hydrogel partially swallowed in acetone. Moreover, Sulfo-Glu hydrogel showed an irreversible response towards mechanical agitation leading to precipitation, similarly to Click-Glu and C_18_-Glu hydrogels.^24^

### Morphology of the xerogels

**Fig. 5:**
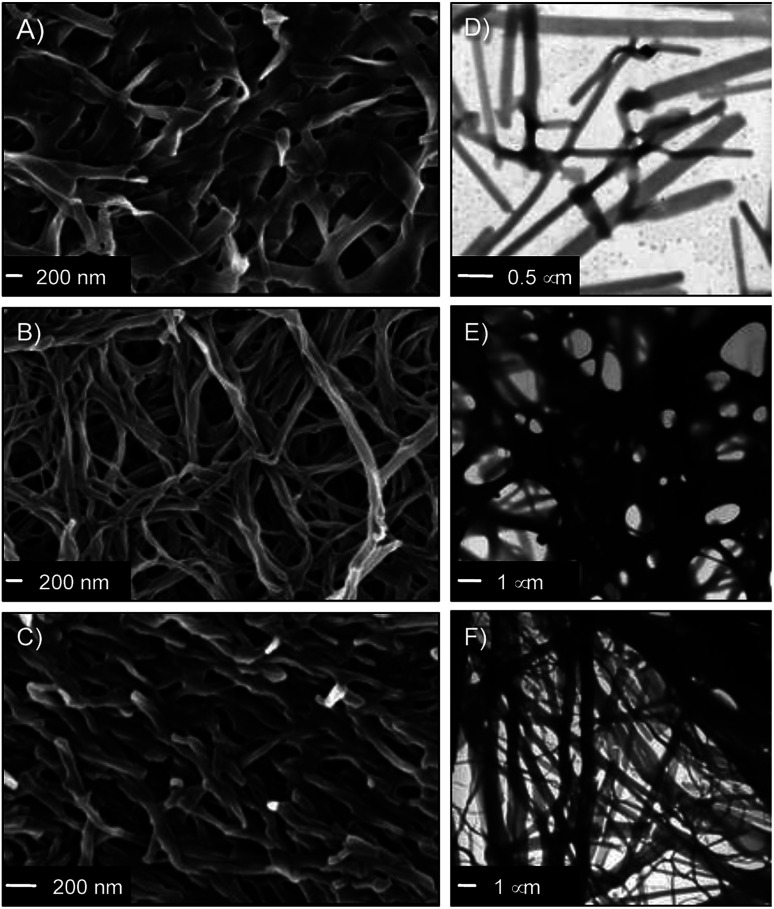
(A–C) Representative FESEM images of Sulfo-Glu xerogels (freeze-dried gels) prepared at the corresponding CGC in (A) water (*c* = 33.3 g L^−1^), (B) acetonitrile (*c* = 3.5 g L^−1^), and (C) toluene (*c* = 13.8 g L^−1^). (D–F) TEM images of the gel samples used to prepare the above described xerogels in (D) water, (E) acetonitrile, and (F) toluene.

The nanostructure of the gel networks was determined by FESEM and TEM imaging of selected xerogels. For comparative purposes, we studied a series of gels prepared in three solvents of different nature (*i.e.* toluene (non-polar), acetonitrile (aprotic polar), water (protic polar)). Sulfo-Glu gels showed a ribbon-type structure of high aspect ratio regardless the solvent (Fig. 5 and S4†), albeit with significant differences with respect to dimensions and organization. For instance, the structure of the Sulfo-Glu hydrogel showed micrometer-length ribbons of *ca.* 150–250 nm in width. However, the ribbons observed in the case of organic solvents were of smaller width (*ca.* 49–114 nm and 54–81 nm in acetonitrile and toluene, respectively). Moreover, the ribbon networks of the gels made in water and toluene were denser than those observed in acetonitrile. This is in good agreement with the different mechanical properties observed by rheology, since the denser structures showed the higher maximum strain values at break (see below). TEM images were also in concordance with the ribbon-type nanostructures observed by FESEM (Fig. 5).

Interestingly, Sulfo-Glu hydrogel displayed a very different morphology compared to the hydrogels made of the previously studied isosteres^24^ at a similar concentration range (*i.e.*C_18_-Glu hydrogel showed nanoalmond-like structures with diameters between 1–5 µm, whereas wrinkled lamellar structures of few µm in length and 100–500 nm in width were observed in the case of Click-Glu hydrogel (Fig. S5†)). These observations are in agreement with a significant influence of the isosteric group in the self-assembly pattern of the gelator. An attempt to rationalize these differences was made using computational studies (see below).

It should be emphasized that the obtained images are representative of the bulk materials. However, although we used two different techniques to identify potential artifacts, the freeze-drying process used for sample preparation could induce morphological changes.^46^ For instance, the hydrogels for FESEM analysis were prepared by freezing in liquid N_2_ and subsequently lyophilized. During this process, water can form ice crystals in such soft materials. Although we did not observe evidences for the formation of ice crystals in our case, interpretation of these images must always be done cautiously.

### Oscillatory rheology

**Fig. 6:**
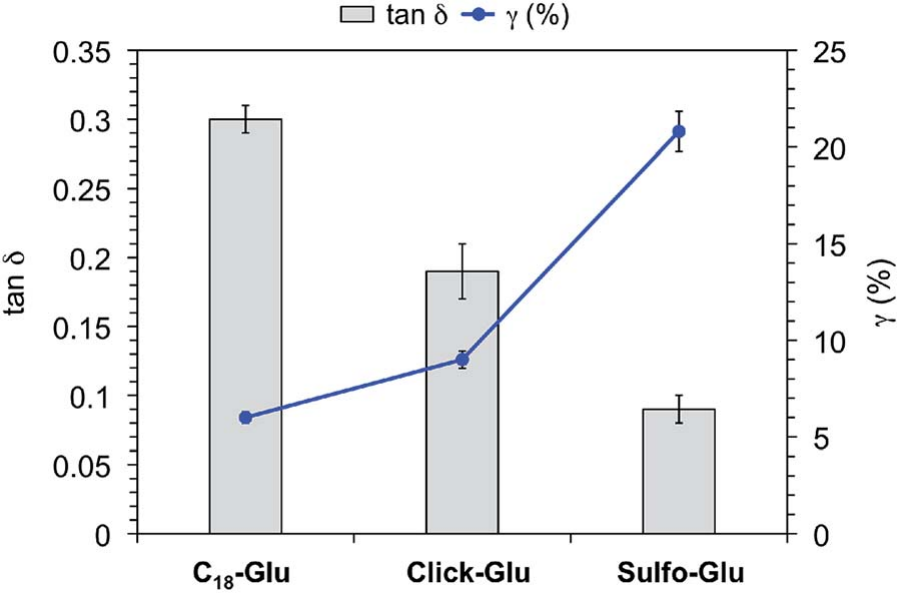
Tan *δ* and strain at break (*γ*) of hydrogels made of C_18_-Glu, Click-Glu and Sulfo-Glu at the CGC.

In order to confirm the viscoelastic nature of the gels and compare their mechanical stabilities towards external shear forces, we performed dynamic frequency sweep (DFS), dynamic strain sweep (DSS), and dynamic time sweep (DTS) measurements of selected gels made in acetonitrile, toluene and water (Fig. S6†). In all cases, the storage modulus *G*′ was approximately one order of magnitude higher than the loss modulus *G*″. Moreover, the maximum strain values at break (*γ*) of the organogels made of Sulfo-Glu were 12% and 10% in acetonitrile and toluene, respectively. These results pointed out that the gels are brittle in nature, likewise those obtained with the other two isosteres. In the case of the hydrogels, DSS experiments confirmed that the hydrogel derived from Sulfo-Glu was less brittle than those made of the other isosteres as suggested by the higher maximum strain at break (*γ*) (*i.e.* 6%, 9%, and 21% for the hydrogels made of C_18_-Glu, Click-Glu, and Sulfo-Glu, respectively) (Fig. 6). The lowest tan *δ* value was observed for the hydrogel made of Sulfo-Glu indicating that this gel acts more elastic and has more potential to store the load rather than dissipating it. These results suggest that the amide-to-sulfonamide isosteric substitution induce, at least to some extent, restrictions against molecular motion of the supramolecular aggregates. Finally, DTS experiments also confirmed the stability of all gels during aging at RT within the viscoelastic regime (*i.e.* 1 Hz frequency, 0.1% strain).

### Self-assembly mode and theoretical studies

In order to get preliminary insights on the aggregation mode of the Sulfo-Glu gelator, we conducted FT-IR studies in different solvents (*i.e.* acetonitrile, toluene, water). A comparative analysis of spectra derived from the solid gelator, gelator solution, and the corresponding xerogels revealed shifted bands suggesting the involvement of non-covalent molecular interactions, mainly hydrogen bonds, with different strength and/or patterns during the formation of the gel networks depending on the solvent nature (Fig. S7†). In previous studies,^24,47^ intermolecular H-bonding between the amide NH-bond and the CO-group of the acid moiety next to the chiral centre of C_18_-Glu was identified as main driving force for its self-assembly in protic solvents leading to multilayered structures. However, both intermolecular H-bonding and intramolecular H-bonding were found to be nearly equally important in organic solvents affording the formation of nanofibers.^24^ The opposite tendency was observed for Click-Glu, suggesting that the isosteric replacement can be used to trigger the formation of desired nanostructures in specific solvents.^24^ In the case of the Sulfo-Glu hydrogel, the *ν*_S=O_ stretching band centered at *ca.* 1300 cm^−1^ was observed regardless the state of the sample (*i.e.* solid gelator, solution and xerogel sample). Partial dissociation of the sulfonamide group would also be compatible with the observed vibration bands. In contrast, the *ν*_C=O_ bands of the carboxylic acid-groups were observed at *ca.* 1740 cm^−1^ for the solid gelator and xerogel samples, whereas the band was shifted to *ca.* 1670 cm^−1^ for the gelator solution. Similarly, *ν*_N–H_ stretching band was observed at *ca.* 3290 cm^−1^ for the solid gelator and xerogel samples, and at *ca.* 2851 cm^−1^ for the gelator solution. However, *ν*_O–H_ and *ν*_C–H_ bands can also overlap in this region, making difficult the identification of the intermolecular interactions based only on the IR pattern. Overall, these results may suggest a strong participation of the carboxylic acid moieties in H-bonding, as well as a preorganization of the gelator molecules in the solid state similarly, at least to some extend, to the hydrogel phase. However, these differences were less evident in the case of organic solvents when comparing solid gelator and gelator solution in toluene or acetonitrile (*e.g.* Δ*ν*_C=O_ < 5 cm^−1^). Overall, these observations support the existence of different gelator arrangements in water (*via* both intra and intermolecular H-bonds) and organic solvents (mainly *via* intermolecular H-bonds). Computational data (see below) clearly reflects the strong intermolecular interactions involving the carboxylic groups in Sulfo-Glu. Also, water (or, in general solvents capable of disrupting the hydrogen bonding in dimers) was found to affect the stabilization energy. This is especially relevant for Sulfo-Glu as hydrogen bonding is particularly relevant.

Additional insights into the self-assembly process were obtained by variable temperature NMR measurements. For instance, a Sulfo-Glu gel made in toluene showed clear shifts of some characteristic peaks depending on the temperature (Fig. S8†). Specifically, the proton signal associated to the sulfonamide group (SO_2_NH̲) shifted 1 ppm upon thermal gel-to-sol phase transition. However, less acidic protons such as that in the α-position (SO_2_NH–CH̲) underwent a smaller shift as its contribution to the stabilization of the gel network is expected to be lower. The inflection point of the *δ* (ppm)–*T* (°C) curves observed at 76.5 ± 0.5 °C matched well with the *T*_gel_ of the material (75.5 ± 1.5 °C) calculated as described in the Experimental section. The area around this melting point was characterized by a fast shift of the corresponding peaks. However, the regions before and after the inflection point, where the shift of the peaks was smaller, corresponded to states dominated by gel and solution phases, respectively. Furthermore, the proton signal associated to the carboxylic group (CO_2_H̲) was not observed below *T*_gel_, and it shifted downfield quickly after this point (*i.e.* from 8.45 to 9.48 ppm within 5 °C), appearing sharp in solution at *ca.* 85–90 °C. This is also in agreement with the FT-IR data suggesting a different rearrangement in solution and in gel phase, where the carboxylic groups play an important role in the generation of a hydrogen-bonded gel network.

In order to understand the nature of the stabilizing intermolecular interactions responsible for the different properties of the gels, we performed a series of theoretical calculations on the structure of different dimers for each isostere (Table S3†). The intermolecular interactions of C_18_-Glu, Click-Glu and Sulfo-Glu were modelled in different solvents by computing two interacting molecules for each compound and solvent. It is worth noting that one of the expected non-covalent interactions may include the long alkyl chains. Thus, the complete structure of the dimer was computed in each case. To include different non-covalent interactions (such as dipole–dipole, π–π staking, dispersion and hydrogen bonding) a number of starting geometries (10–20 initial structures) were considered by modifying the relative position between the two molecules. Different interaction possibilities were considered for each case. These structures were then fully optimized to obtain 8–10 minima (in some cases, different initial guesses converged to the same minimum).

**Fig. 7:**
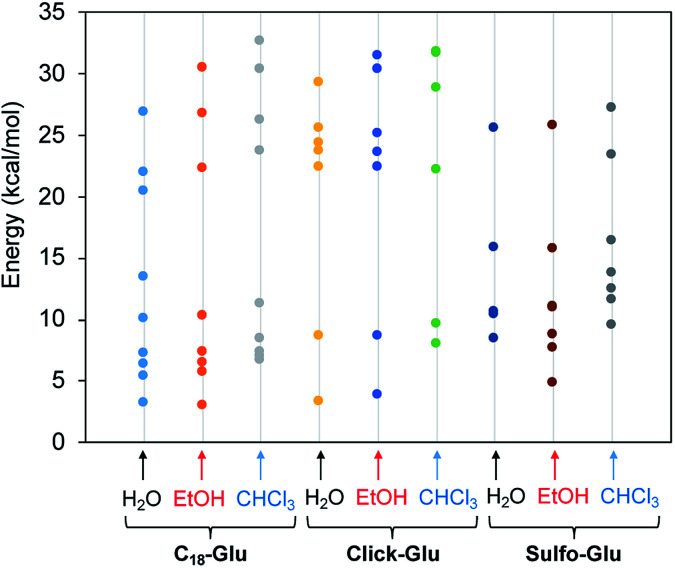
Stabilization energy of dimers for the three isosteres (C_18_-Glu, Click-Glu and Sulfo-Glu) in water, ethanol and chloroform with respect to separated molecules in each environment.

**Fig. 8:**
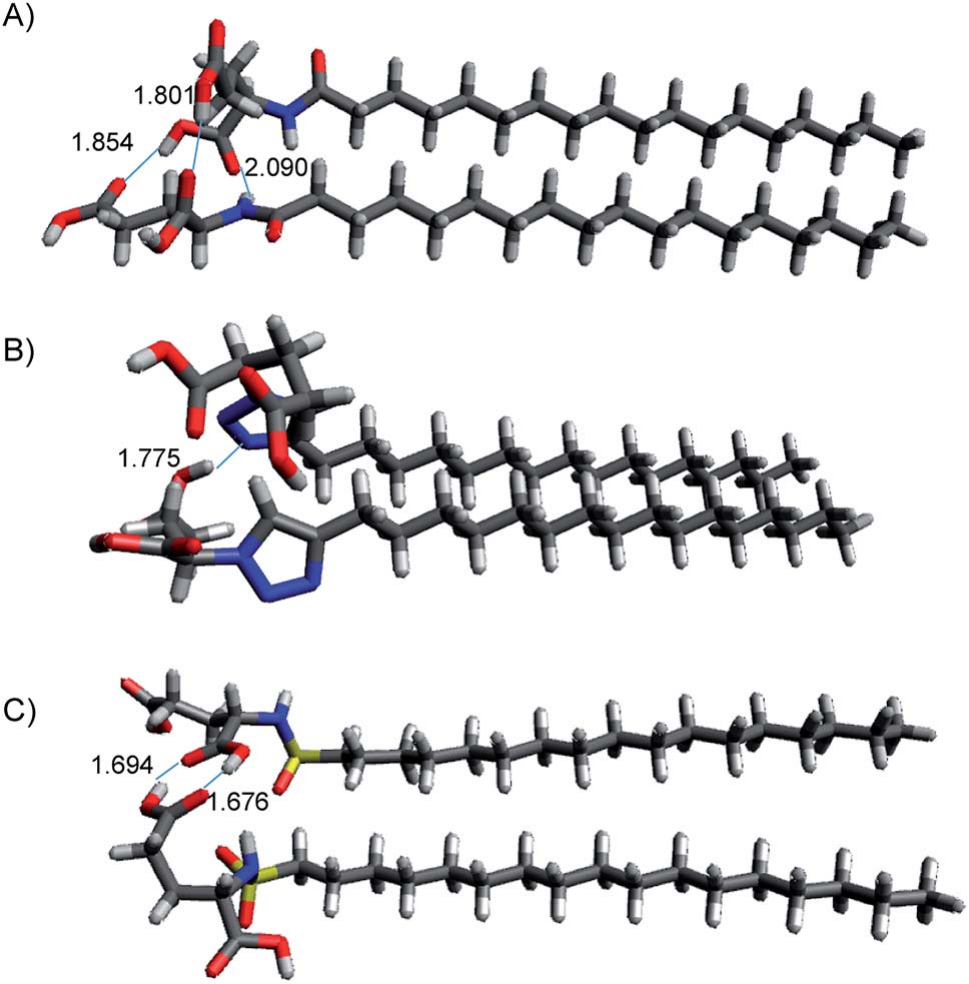
Structures of lowest energy minima for (A) C_18_-Glu, (B) Click-Glu and (C) Sulfo-Glu in chloroform. Hydrogen bonds are represented by dotted lines, distance in Å.

Fig. 7 represents the relative energies obtained for each minimum for the different compounds in the solvents under study. In all cases the energy is referenced to the two separated molecules to account for the stabilization energy. As it can be seen, two sets of structures were found in all cases. One type of minima implies stabilization energies of 20–30 kcal mol^−1^. The second set of geometries implies stabilization energies around 5–15 kcal mol^−1^ (see Fig. 8 for representative geometries). Not surprisingly due to the additive effects of the different components of the stabilization energy (namely, dispersion forces, hydrogen bonding, dipole–dipole, *etc.*), the set of structures with higher stabilization energies implies both hydrogen bonding between the polar side of the molecules and interaction between the alkyl chains.

In contrast, in the cases in which the alkyl chains cannot interact, the stabilization energy is clearly lower, as it happens in the second set of minima. Although, the results for C_18_-Glu, Click-Glu and Sulfo-Glu are similar, in the case of Sulfo-Glu the difference between the two sets of structures is smaller. This could imply a minor preference for the structures with parallel alkyl chains. As a general trend, the polarity of the solvent affects the stability of the different minima. Chloroform provides for the three isosteres higher stabilization energies for the set of minima with higher values (see Fig. 7, columns for CHCl_3_). Again, this effect is smaller for Sulfo-Glu.

The most stable computed geometries for each isostere in chloroform are shown in Fig. 8. Several types of interactions could be observed is the different computed minima and are represented in the selected geometries. Different hydrogen bonds are available for the three types of compounds. When present, this type of interaction greatly contributes to the formation of stable dimers. In addition, interaction between alkyl chains is also evident for most structures when the orientation of the two molecules allows it. These two interactions were found to be the most important ones, even when other possibilities arose. Especially relevant in terms of stabilization are those geometries in which both stabilizing interactions can operate at the same time. Alternatively, the ring in Click-Glu could allow for π–π stacking or H⋯π interactions. However, these options were found to only slightly contribute in some geometries and were comparatively minor compared to other interactions. The effect of the computed solvent was found to cause minor alterations in the geometries for each stable minimum. This suggests that these molecules can interact in different orientations in diverse environments. While the relative stability for each dimer would depend on the polarity of the environment and the available interactions for the compound, these results confirm the experimental trend found for C_18_-Glu, Click-Glu and Sulfo-Glu to form gels in a quite different range of solvents.

### *In vitro* cytotoxicity studies

In order to preliminary evaluate the potential of the hydrogels for biomedical applications, we performed comparative *in vitro* cytotoxicity studies of the isosteric gelators in HeLa human tumor cells using the MTT assay. This colorimetric assay discerns between viable cells, which convert MTT into purple formazan, and dead cells, which do not react with MTT. Formazan is then accumulated inside cells and its quantity, which gives the number of viable cells, is measured by absorbance at 560 nm.

**Fig. 9:**
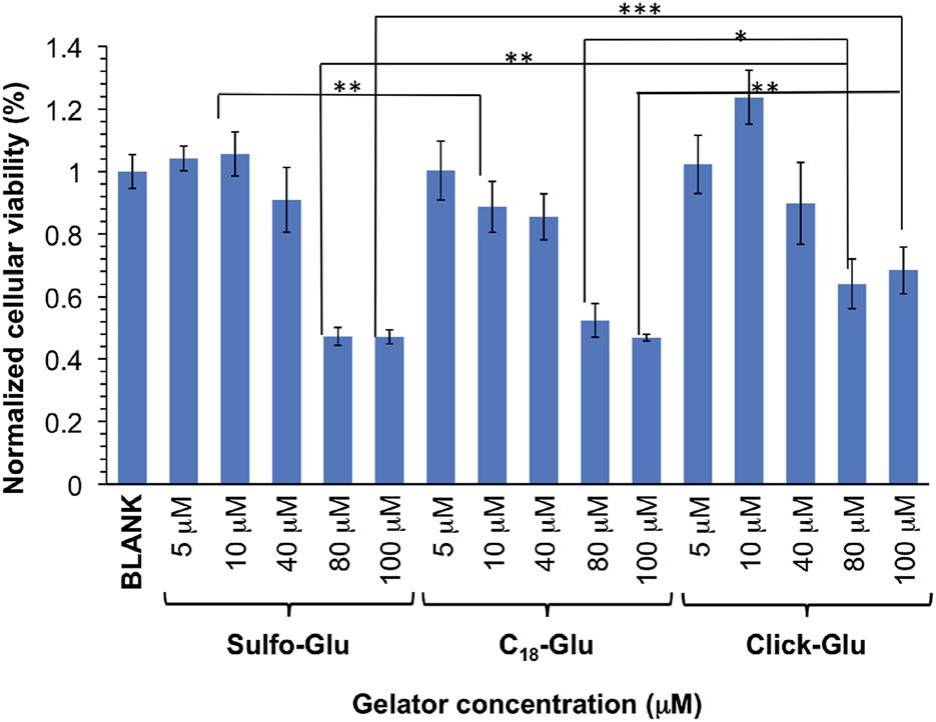
*In vitro* cell viabilities of isosteric gelators at different concentrations within the range 5–100 µM in HeLa cells (*n* = 6, error bars represented the standard deviation).

The cellular viability percentage was studied for 8 h at several gelator concentrations that ranged from 5 to 100 µM and it was compared to the negative control (blank) in which HeLa cells were incubated in the absence of the aforementioned gelators (Fig. 9). The results indicated good cell viabilities below 80 µM (over 85%) and showed above 45% cell viability at concentration between 80 µM and 100 µM. The Click-Glu analogue displayed a slight increase of viability (over 65%) at high concentration (80 µM and 100 µM) compared to Sulfo-Glu. Significance analyses were carried out at low and high concentration. Cellular viabilities values obtained with the Sulfo-Glu gelator were significant compared to C_18_-Glu (**ρ < 0.001) at 10 µM. Cellular viability values for Click-Glu were found significant compared to Sulfo-Glu (**ρ < 0.001) and C_18_-Glu (*ρ < 0.05) gelators at 80 µM. This significance was also found at 100 µM for Sulfo-Glu (***ρ < 0.0001) and C_18_-Glu (**ρ < 0.001). Overall, the results suggest that Sulfo-Glu has low cytotoxicity and is suitable to be used in cell culture.

### Antibiotic release studies

In our previous studies,^24^ both C_18_-Glu and Click-Glu hydrogels were found to be suitable carriers for the sustained release of several bioactive molecules with different hydrophilic/hydrophobic balance (*i.e.* antibiotic vancomycin; anticancer drugs methotrexate, camptothecin and flutamide). The results of these studies indicated different release rates depending on the isostere used to prepare the hydrogels.^24^ In this work, we have studied and compare the encapsulation and release of vancomycin using Sulfo-Glu hydrogel as carrier.

**Fig. 10:**
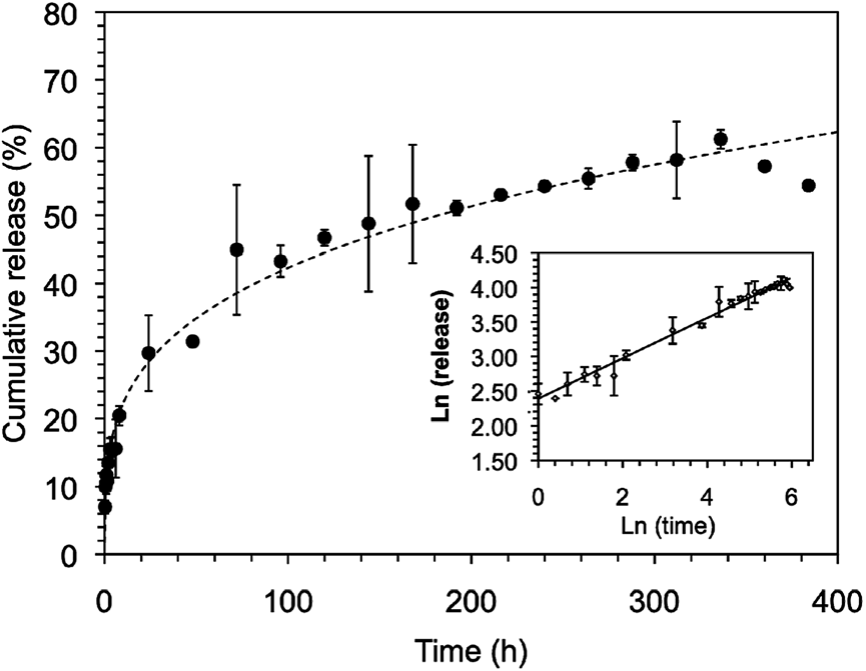
Release profiles of vancomycin in PBS buffer from the hydrogel made of Sulfo-Glu (*i.e.* [Sulfo-Glu] = 1.3 × 10^−4^ M; [vancomycin] = 1.38 × 10^−3^ M). Dash-line: Korsmeyer–Peppas fitting. Inset: Ln–Ln plot.

Stable hydrogels were prepared in PBS using Sulfo-Glu and a vancomycin concentration of 1.38 × 10^−3^ M in order to compare the release profiles with those obtained with the previous isosteric gelators.^24^ No significant differences in *T*_gel_ (55.5 ± 1.5 °C) were observed between the pristine hydrogel and the vancomycin-loaded hydrogel. The drug release rate over 14 days revealed a sustained release with no burst effect and a rate constant *k*_obs_ = 1.2 ± 0.1 × 10^−2^ h^−1^. The release profile was well adjusted using the Korsmeyer–Peppas model showing a quasi-Fickian diffusion mechanism (*i.e.* release exponent (*n*) < 5, non swellable matrix-diffusion) (Fig. 10). The observed *k*_obs_ value was comparable to those obtained in the C_18_-Glu hydrogel (*k*_obs_ = 1.5 ± 0.2 × 10^−2^ h^−1^) and smaller than the constant obtained with the Click-Glu hydrogel (*k*_obs_ = 4.3 ± 0.3 × 10^−2^ h^−1^).^24^ After 2 weeks, the drug diffusion from the Sulfo-Glu hydrogel reached a plateau at *ca.* 60% of the initial drug concentration, comparable to that obtained from C_18_-Glu hydrogel (*ca.* 56% of drug release) but inferior to that from Click-Glu hydrogel (*ca.* 90% of drug release).^24^ The absence of burst release suggested a proper incorporation of the drug in the hydrogel matrix. Although such prolonged vancomycin delivery is desired in order to eliminate the need for multiple dosing, faster release using this type of hydrogels can also be achieved upon decreasing the gelator concentration due to the decrease of the crosslink density of the network.^24^ The differences observed in the *k*_obs_ of the three cases are probably due mainly to changes in the microstructure of the gels and the interaction patterns between solvent–gelator, gelator–gelator and gelator/fibril-drug. Thus, different diffusion properties of the drug through these hydrogels are ultimately governed by the isosteric gelator structure.

**Fig. 11:**
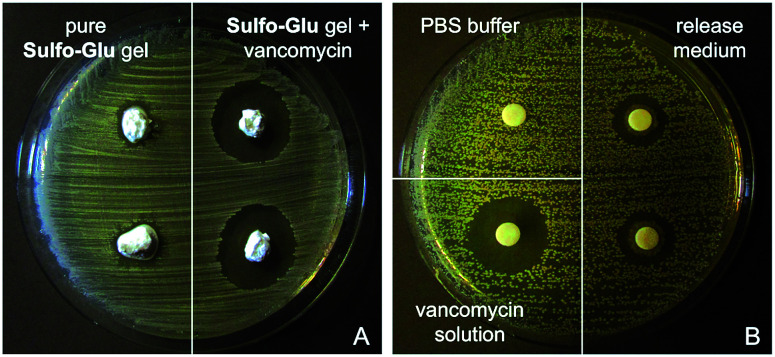
(A) Kirby–Bauer test against *Staphylococcus aureus* using Sulfo-Glu hydrogel samples with and without vancomycin. (B) Kirby–Bauer test against *Staphylococcus aureus* using filter paper soaked with medium from drug release test, PBS buffer and an aqueous vancomycin solution (*c* = 2 mg mL^−1^).

As expected, Sulfo-Glu gels loaded with vancomycin also showed antimicrobial activity against the Gram-positive bacteria *Staphylococcus aureus*^48^ in the Kirby–Bauer test. Large inhibition zones (*∅* = 22.5 ± 0.5 mm) were formed around the vancomycin-containing Sulfo-Glu hydrogel samples, whereas the bacteria grew almost to the edge after 24 h in the presence of control hydrogels (*i.e.* hydrogels without antibiotic) (Fig. 11). These results indicate a lack of antibacterial activity of the sulfonamide gelator.^49^ A positive control (*i.e.* aqueous vancomycin solution (*c* = 2 mg mL^−1^), inhibition zone *∅* = 20 mm) and a negative control (pure PBS buffer) were also employed in this study. The results indicated that only part of the drug diffused from the gel, since the positive control being 3 times less concentrated showed comparable antibacterial effect. This is in good agreement with the results obtained in the drug release experiments. Moreover, the antimicrobial activity displayed by the solution taken from the drug release experiment after 24 h was tested with positive results in the inhibition test (zone *∅* = 14.5 ± 0.5 mm). In our previous studies, vancomycin-containing C_18_-Glu and Click-Glu hydrogels showed inhibition zones of 18 mm and 21.5 mm in diameter, respectively.^24^ Thus, the antimicrobial activity of the antibiotic-loaded hydrogels can be tuned to some extent based on the isosteric gelator used, with inhibition zones ranging from *ca.* 18 to 23 mm after 24 h.

Released vancomycin concentrations were found to be above the reported minimal inhibition concentration (MIC90) for *Staphylococcus aureus* (MRSA) (=2 µg mL^−1^)^50^ at all-time points of the release study (*i.e.* the lowest vancomycin concentration released was *ca.* 126 µg mL^−1^, which corresponds to 63-fold of the MIC90). The bactericidal activity relative to the positive control was found to decrease slightly (*ca.* 10% reduction of the inhibition zone after 2 weeks), probably due to partial hydrolysis of the antibiotic.^32^

## Conclusions

The foregoing results indicate that the isosteric substitution of an amide in a LMW gelator by a sulfonamide moiety can be used for fine-tuning the properties of the corresponding supramolecular gels. In comparison with 1,2,3-triazoles as amide isosteres, sulfonamides allow to expand the gelation ability and enhance significantly key properties such as gelation concentration, gel-to-sol transition temperature, mechanical properties, and gelation kinetics in several organic solvents and water. Different stabilizing interactions were computed for each compound to understand the gelation properties. Moreover, the amide-sulfonamide isosteric replacement also influences the morphology of the gel networks as well as the release rate of encapsulated drugs. The potential applications of stimuli-responsive gels^17–19^ range from drug delivery,^51^ photon upconverting materials,^52^ optoelectronics,^53^ energy,^47^ and sensors,^54^ among others.

In general, the results of this study place sulfonamide groups as useful isosteres of amides that, together with 1,2,3-triazoles, can be used to develop supramolecular gels with new properties starting from known amide-containing LMW gelators. Finally, the higher chemical robustness of 1,2,3-triazoles and sulfonamides compared to amides may also give to this strategy practical importance for the synthesis of new functional materials in other research fields.

## Conflicts of interest

There are no conflicts to declare.

## Supplementary Material

RA-010-D0RA00943A-s001
