# Supplementary material for: Sulfonamide as amide isostere for fine-tuning the gelation properties of physical gels
Source: RSC Adv. 2020 Mar 20;10(19):11481–92. doi: 10.1039/d0ra00943a (PMC9050504; doi:10.1039/d0ra00943a)
Supplement: RA-010-D0RA00943A-s001 [file RA-010-D0RA00943A-s001.pdf]

Electronic Supplementary Material (ESI) for RSC Advances.
This journal is © The Royal Society of Chemistry 2020

Supporting Information

Sulfonamide as amide isostere for fine-tuning the gelation properties of physical gels

Juan V. Alegre-Requena,[a,b] Santiago Grijalvo,[c,d] Diego Sampedro,[e] Judith Mayr,[a] César Saldías,[f] José Juan Marrero-Tellado,[g,h] Ramón Eritja,[c,d] Raquel P. Herrera*[b] and David Díaz Díaz*[a,g,h]

[a] *Institut für Organische Chemie, Universität Regensburg, Universitätsstr. 31, 93053 Regensburg, Germany.*
E-mail: David.Diaz@chemie.uni-regensburg.de
[b] *Laboratorio de Organocatálisis Asimétrica, Departamento de Química Orgánica, Instituto de Síntesis Química y Catálisis Homogénea (ISQCH), CSIC-Universidad de Zaragoza, Pedro Cerbuna 12, 50009 Zaragoza, Spain*
E-mail: raquelph@unizar.es
[c] *Biomedical Research Networking Centre in Bioengineering, Biomaterials and Nanomedicine (CIBER-BBN), Jordi Girona 18-26, 08034 Barcelona, Spain*
[d] *Institute of Advanced Chemistry of Catalonia (IQAC-CSIC), Jordi Girona 18-26, 08034 Barcelona, Spain*
[e] *Departamento de Química, Universidad de La Rioja, Madre de Dios, 51, 26006 Logroño, Spain*
[f] *Departamento de Química Física, Facultad de Química y de Farmacia, Pontificia Universidad Católica de Chile, Macul, 7820436 Santiago, Chile*
[g] *Departamento de Química Orgánica, Universidad de La Laguna, Avda. Astrofísico Francisco Sánchez, 38206 La Laguna, Tenerife, Spain*
E-mail: ddiazdiaz@ull.edu.es
[h] *Instituto Universitario de Bio-Orgánica Antonio González, Universidad de La Laguna, Avda. Astrofísico Francisco Sánchez 2, 38206 La Laguna, Tenerife, Spain*

Contents List

		page
1.	Tabular data of gelation ability and gel properties ……………………………….	S3
2.	Concentration dependence of gelation time and T_{gel} …………………………...	S5
3.	External stimuli-responsive behavior …………………………………………….	S6
4.	Additional electron microscopy images …………………………………………..	S7
5.	Rheological measurements ……………………………………………………….	S9
6.	FT-IR spectra of gel and xerogel materials ……………………………………….	S11
7.	Variable temperature NMR experiments …………………………………………	S15
8.	Cartesian coordinates ……………………………………………………………..	S17
9.	NMR spectra ……………………………………………………………………...	S25

1. Tabular data of gelation ability and gel properties

Table S1 Gelation ability and typical gel properties of **Sulfo-Glu** in H_2O and diverse organic solvents.[a]

Solvent	CGC (g/L)	Gelation time (min)	T_{gel} (ºC)	Appearance
CH_2Cl_2	32.5±3.9	2.77±0.10	50.7±2.4	OG
$CHCl_3$	45.0±5.0	1.95±0.18	57.0±2.2	OG
CH_3CN	3.5±0.2	20.8±0.3	35.5±0.5	OG
H_2O	33.3	>12h	55.5±1.5	OG
MeOH		PG at ≤ 200 g/L		
Glycerol	5.5±0.6	15±2	32	OG
Et_2O		PG at ≤ 200 g/L		
Hexane		PG at ≤ 200 g/L		
Xylene	23.6±1.4	3.58±0.41	50.5±1.5	OG
Benzene	18.2	0.78±0.11	66.7±1.8	OG
Toluene	13.8±0.5	7.37±0.19	72±2	OG
EtOH		PG at ≤ 200 g/L		
*i*PrOH		PG at ≤ 200 g/L		
2-Butanol		PG at ≤ 200 g/L		
1-Hexanol		PG at ≤ 200 g/L		
DMSO		Soluble		
DMF		Soluble		
THF		PG at ≤ 200 g/L		
AcOEt	47.2±2.8	6.29±0.21	42	OG
Acetone		PG at ≤ 200 g/L		
Chlorobenzene	26.8±1.8	85±5	58.5±0.5	TLG/OG
Benzonitrile	47.2±2.8	4.20±0.33	58±1	TLG/OG

[a] Error values reported as STDV were estimated from at least two randomized experiments. Abbreviations: CS = clear solution resulting after heating a mixture of the compound and the corresponding solvent; I = insolubility of the compound in the corresponding solvent; room temperature ionic liquid); OG = opaque gel; P = precipitation of the compound from isotropic solutions of **Sulfo-Glu** and the corresponding solvent; PG = partial gel (phase separation between gel and sol after time); TLG = translucent gel.

Table S2 Gel properties of **Sulfo-Glu** in H_2O and diverse organic solvents at the maximum CGC of the three isosteres.[a]

Solvent	Concentration (g/L)	Gelation time (min)	T_{gel} (ºC)
CH_2Cl_2	180	28±6	72±2
$CHCl_3$	100	46±4	62±1
CH_3CN	110	23±5	71±1
Glycerol	20	239±16	61±1
Xylene	95	51±1	83±2
Benzene	200	21±3	80±1
Toluene	135	37±5	82±1

[a] Error values reported as STDV were estimated from at least two randomized experiments.

2. Concentration dependence of gelation time and T_{gel}

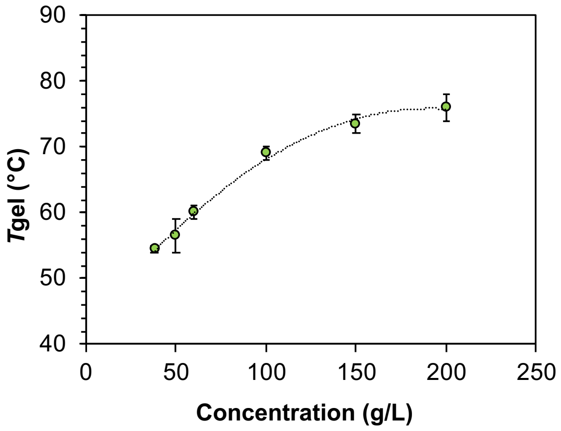

Fig. S1 T_{gel} evolution of **Sulfo-Glu** hydrogels depending on gelator concentration.

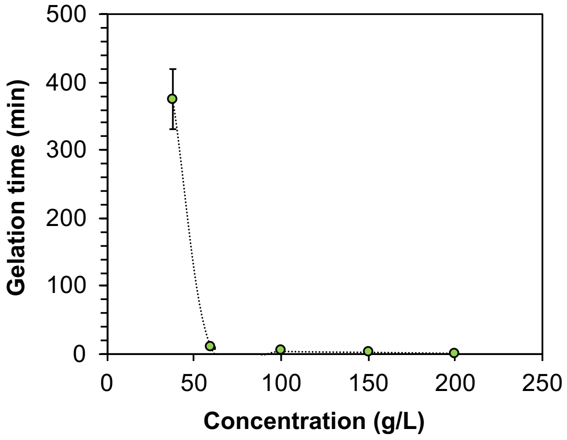

Fig. S2 Gelation time of **Sulfo-Glu** hydrogels depending on the gelator concentration.

3. External stimuli-responsive behavior

A)

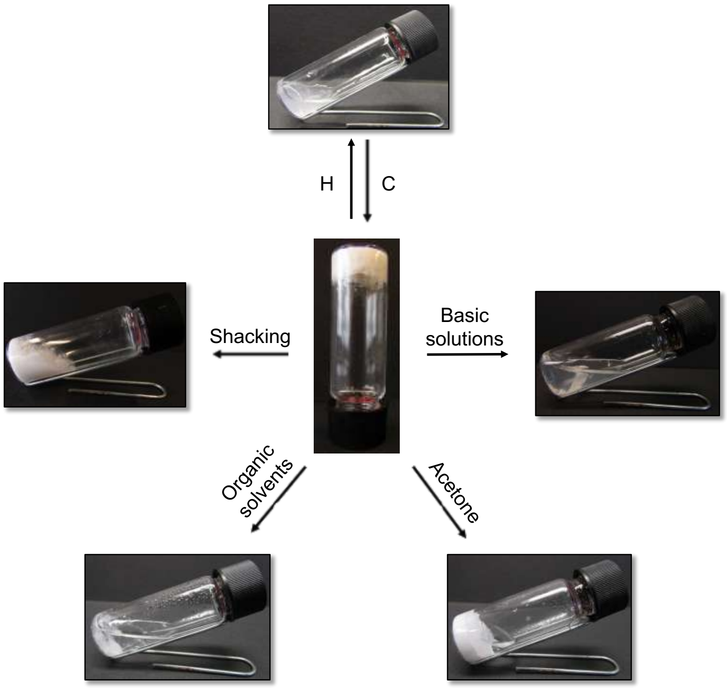

B)

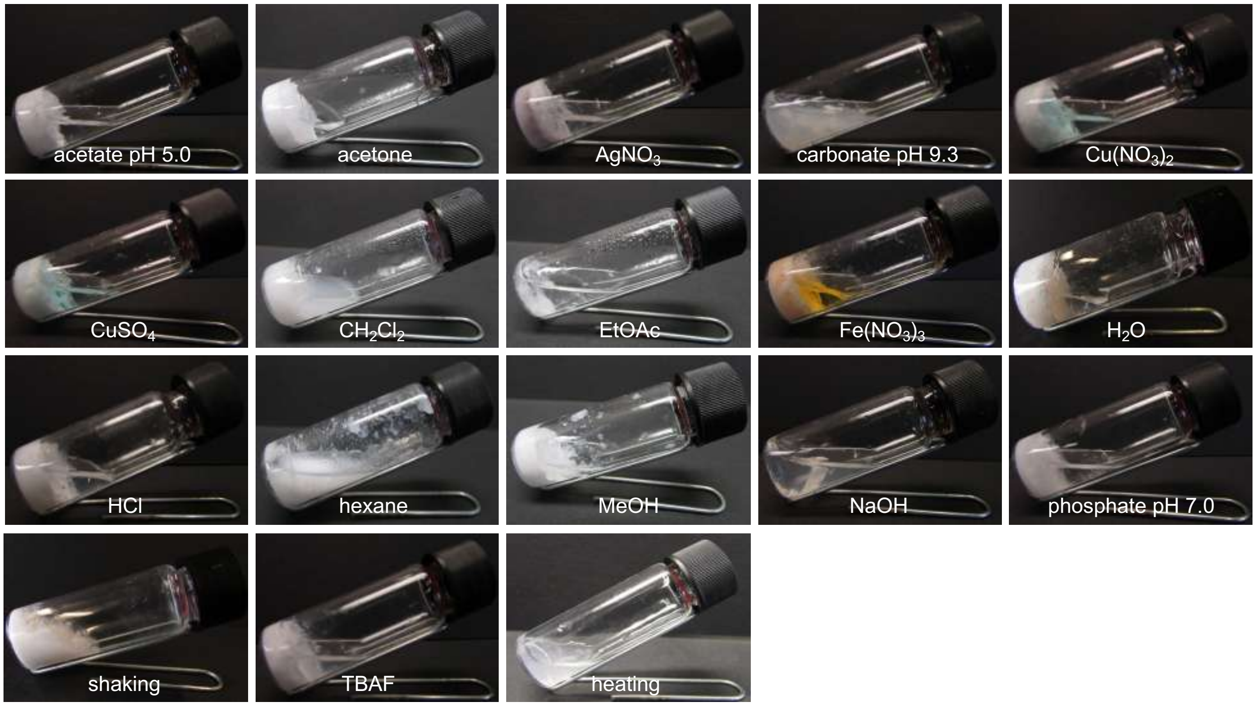

Fig S3 A) Representative diagram showing the external stimuli response map of **Sulfo-Glu** hydrogels. Abbreviations: C = cooling; H = heating. B) Appearance of **Sulfo-Glu** hydrogels in the presence of different additives and stimuli.

4. Additional electron microscopy images

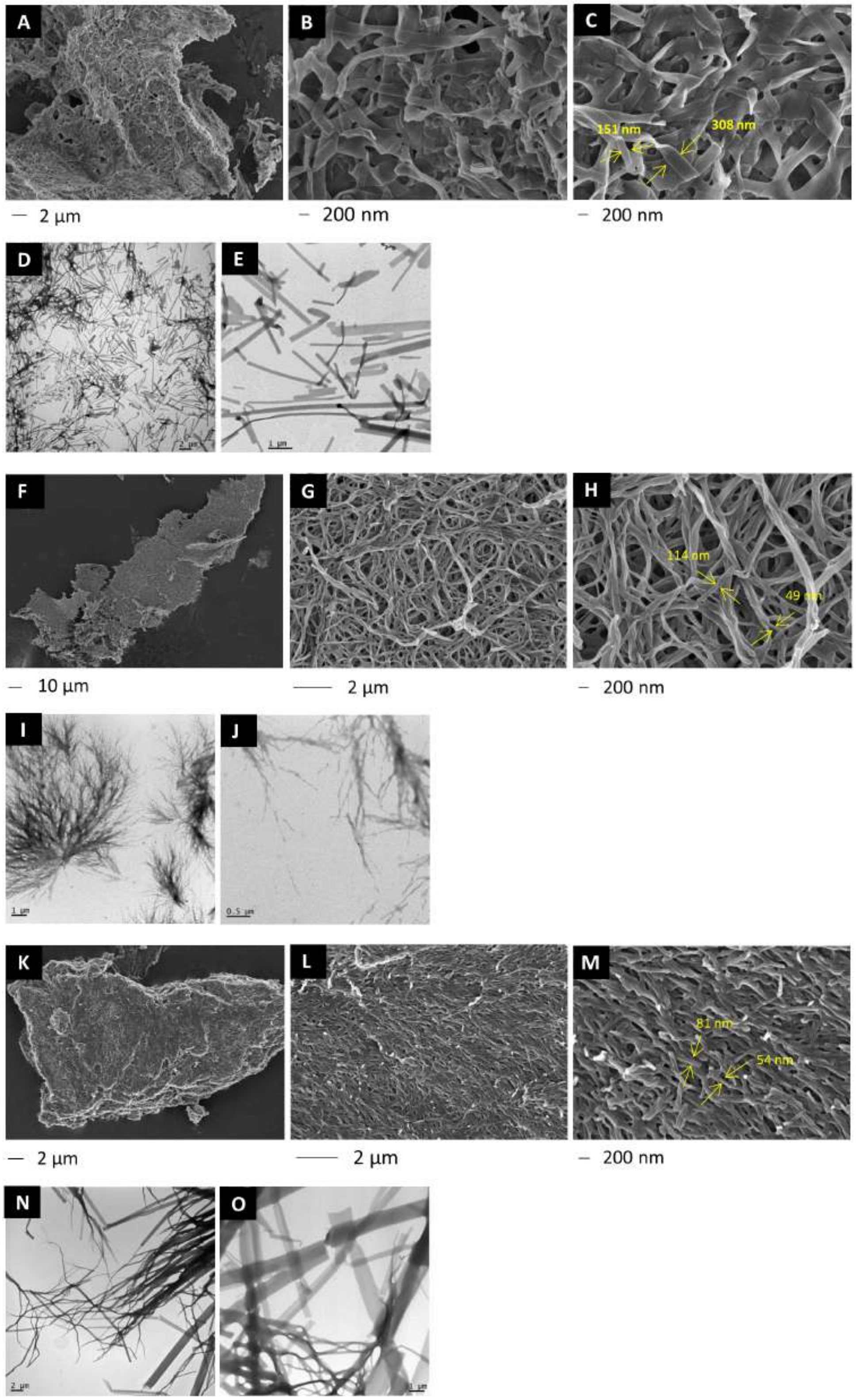

Fig. S4 FESEM (**A**-**C**, **F**-**H** and **K**-**M**) and TEM (**D**-**E**, **I**-**J** and **N**-**O**) images of materials derived from **Sulfo-Glu** at the CGC of gels made in different solvents. **A**-**E**: H_2O (33.3 g/L); **F**-**J**: CH_3CN (3.5 g/L); **K**-**O**: toluene (13.8 g/L).

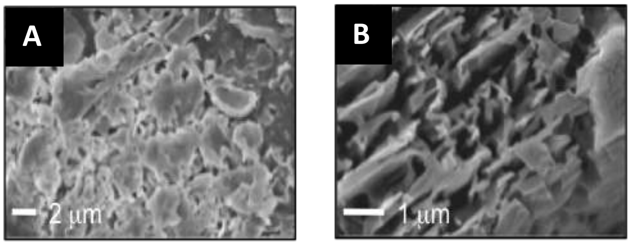

Fig. S5 FESEM images of the xerogels prepared from the hydrogels made of A) **C_{18}-Glu** (c = 25 g L^{-1}) and B) **Click-Glu** (c = 25 g L^{-1}).

We would like to acknowledge the use of Servicio General de Apoyo a la Investigación-SAI and the Laboratorio de Microscopías Avanzadas at Instituto de Nanociencia de Aragón, Universidad de Zaragoza, for FESEM and TEM imaging.

4. Rheological measurements

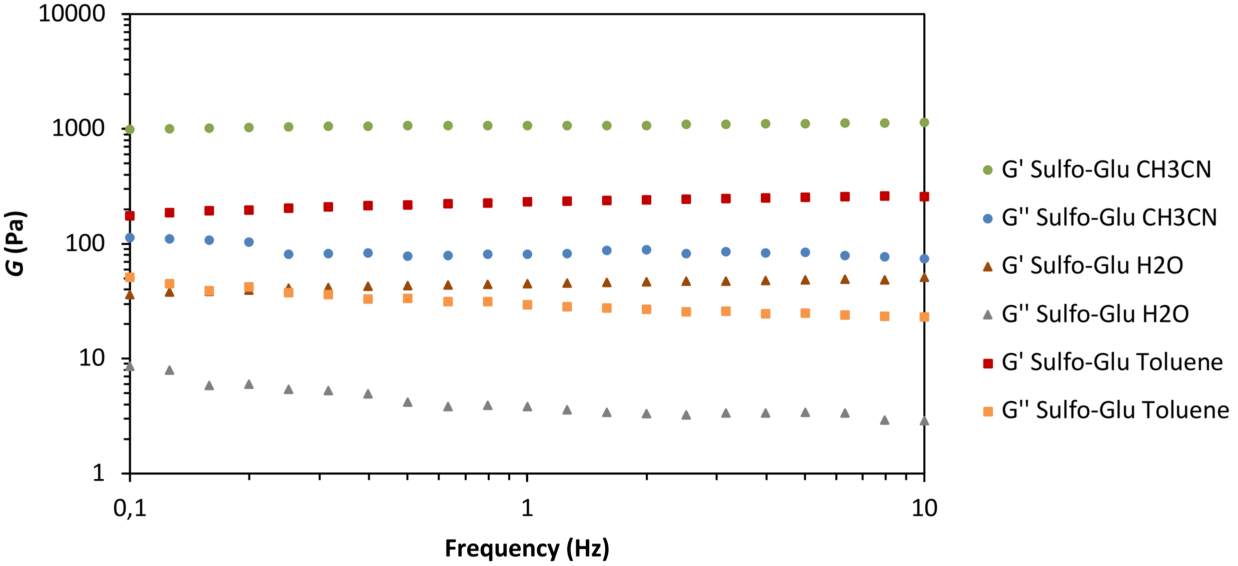

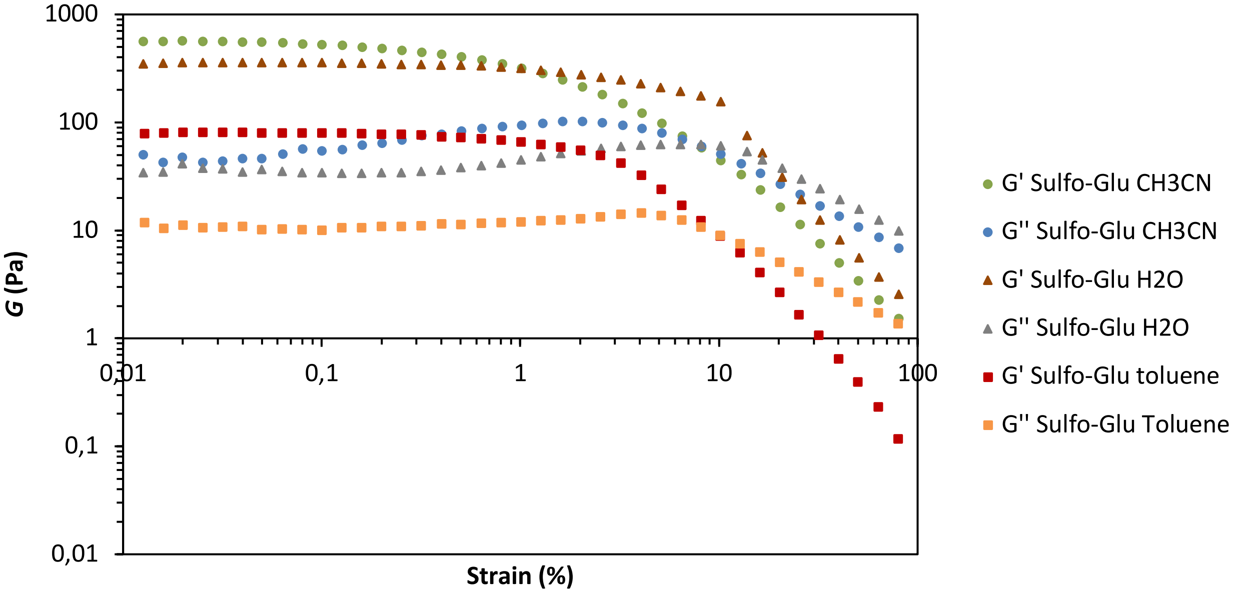

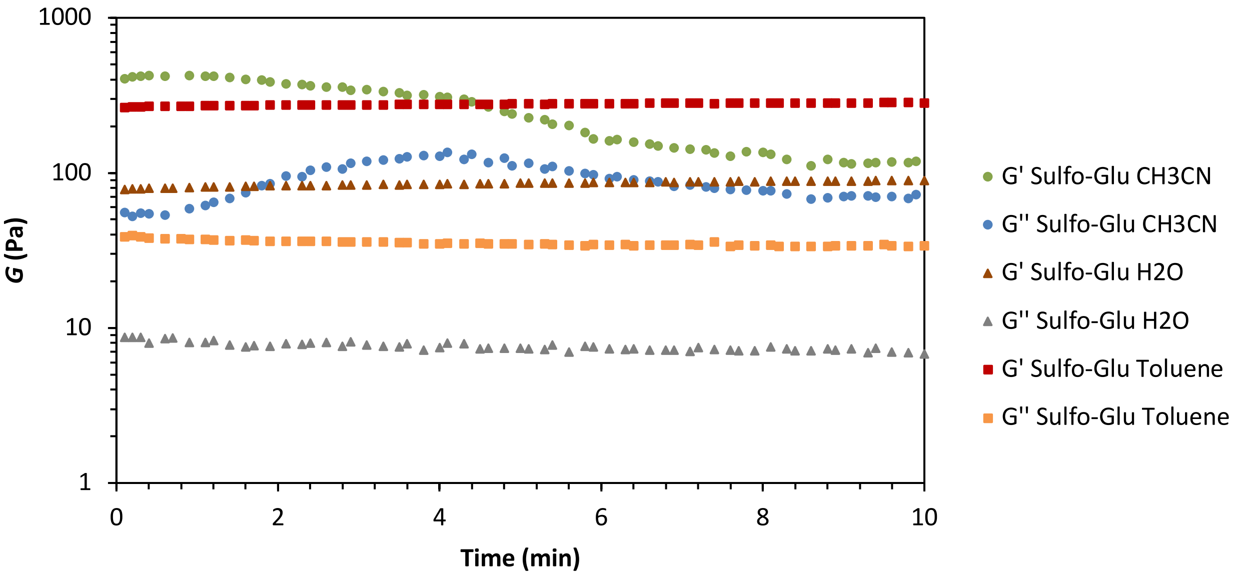

Fig. S6 Oscillatory rheological experiments of model gels prepared from **Sulfo-Glu** in water and toluene at 33.3 g/L. *Top*: DFS-plots. *Middle*: DSS-plots. *Bottom*: DTS-plots.

6. FT-IR spectra of gel and xerogel materials

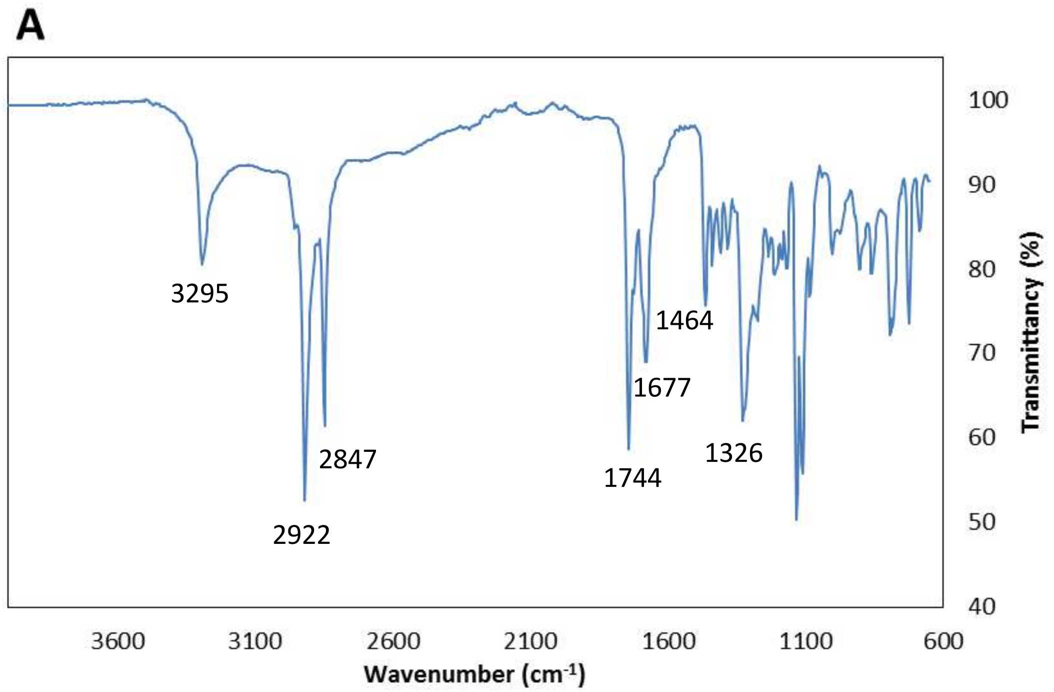

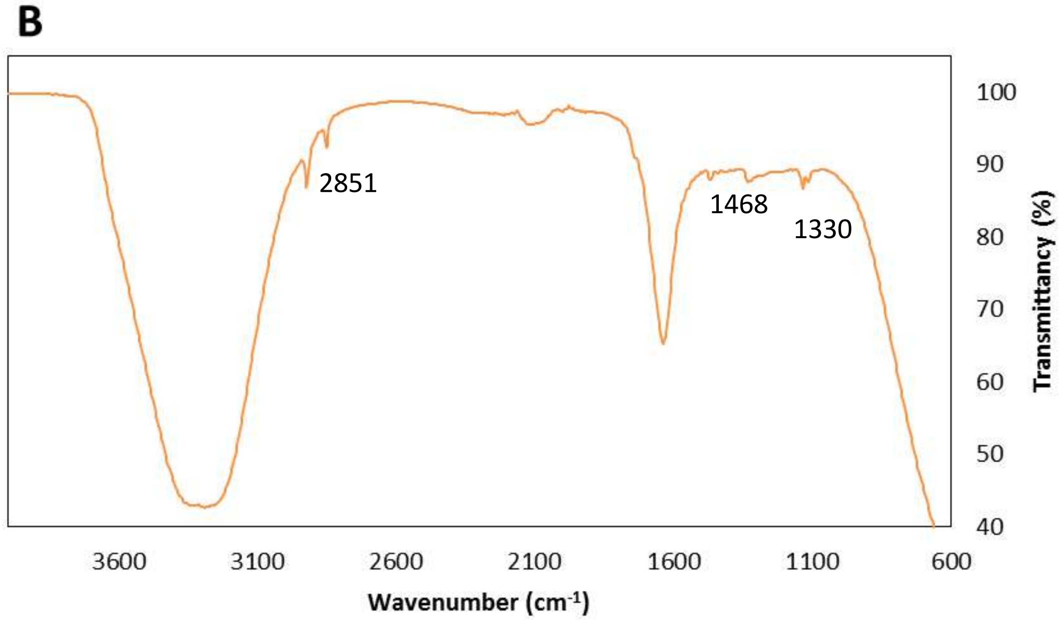

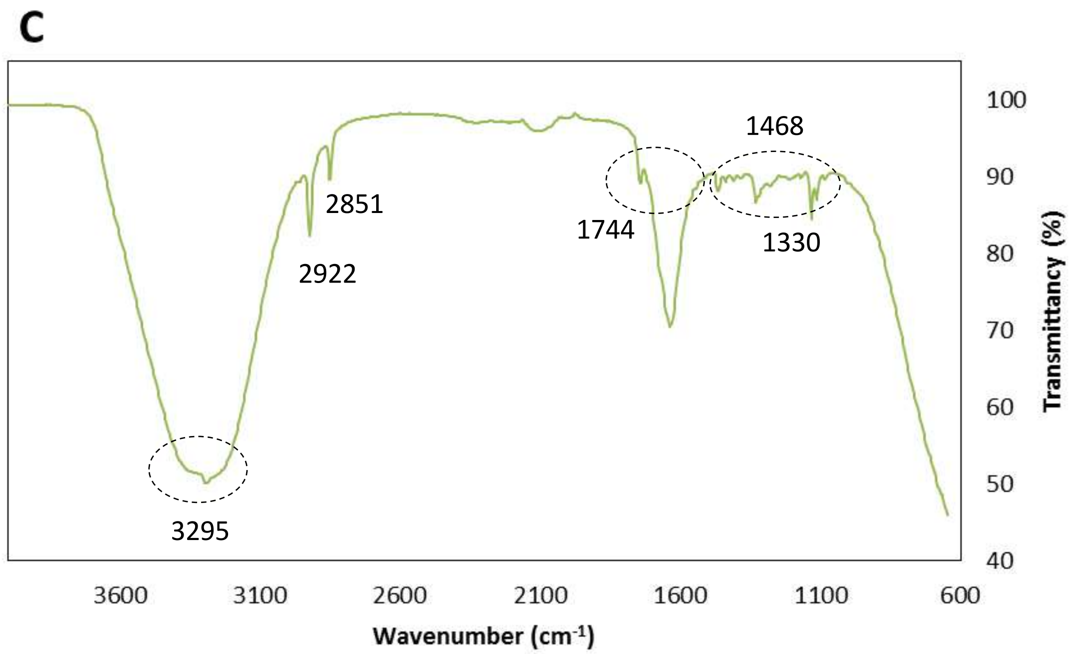
C
100
90
80
70
60
50
40
Transmittancy (%)
1468
2851
1744
1330
2922
3295
3600
3100
2600
2100
1600
1100
600
Wavenumber (cm-1)

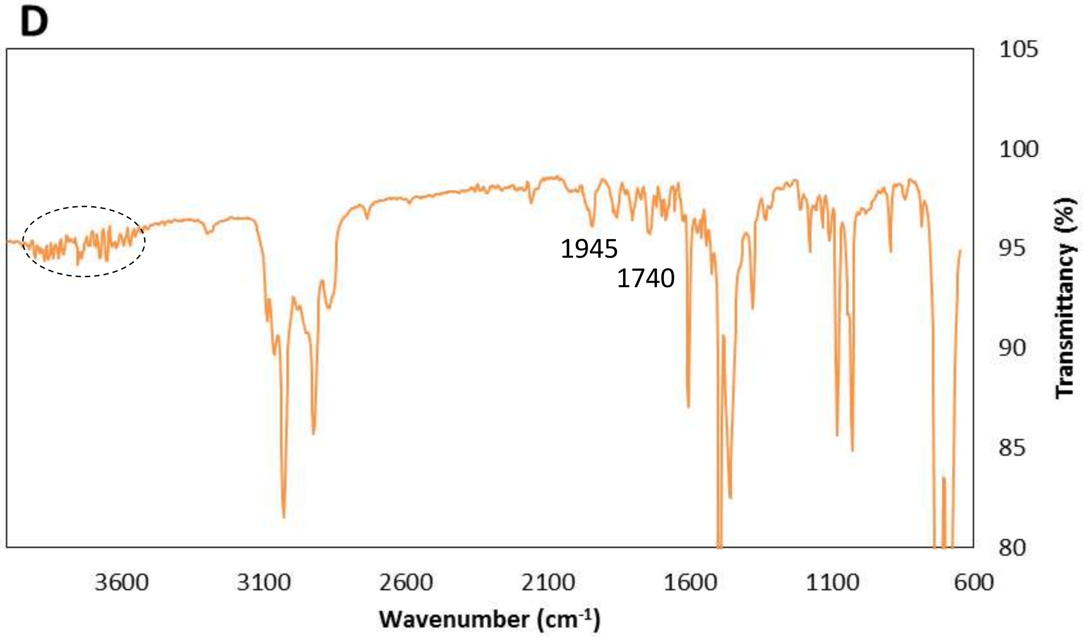
D
105
100
95
90
85
80
Transmittancy (%)
1945
1740
3600
3100
2600
2100
1600
1100
600
Wavenumber (cm-1)

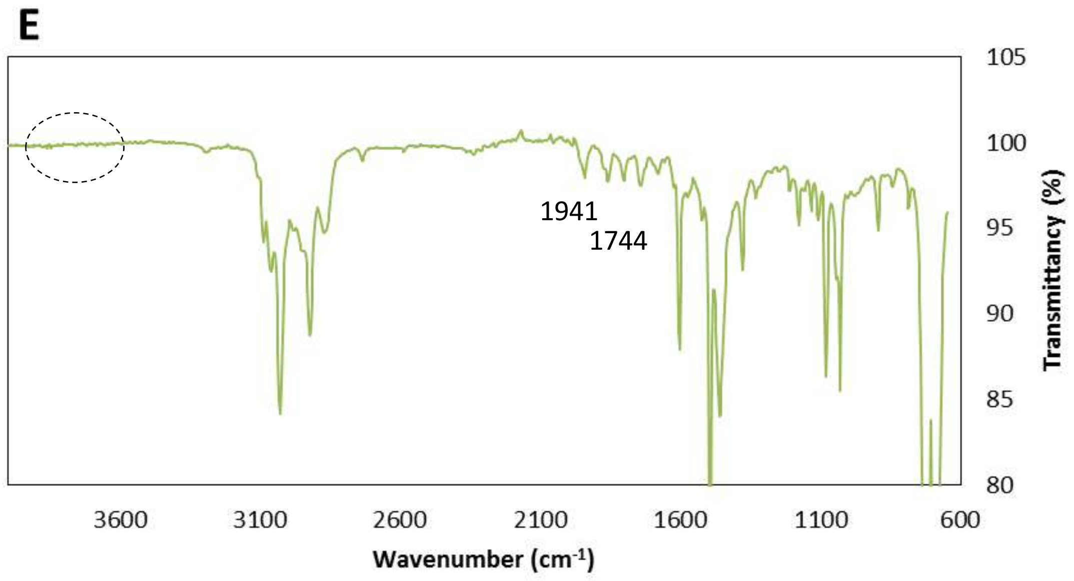
E
1941
1744
105
100
95
90
85
80
Transmittancy (%)
3600
3100
2600
2100
1600
1100
600
Wavenumber (cm-1)

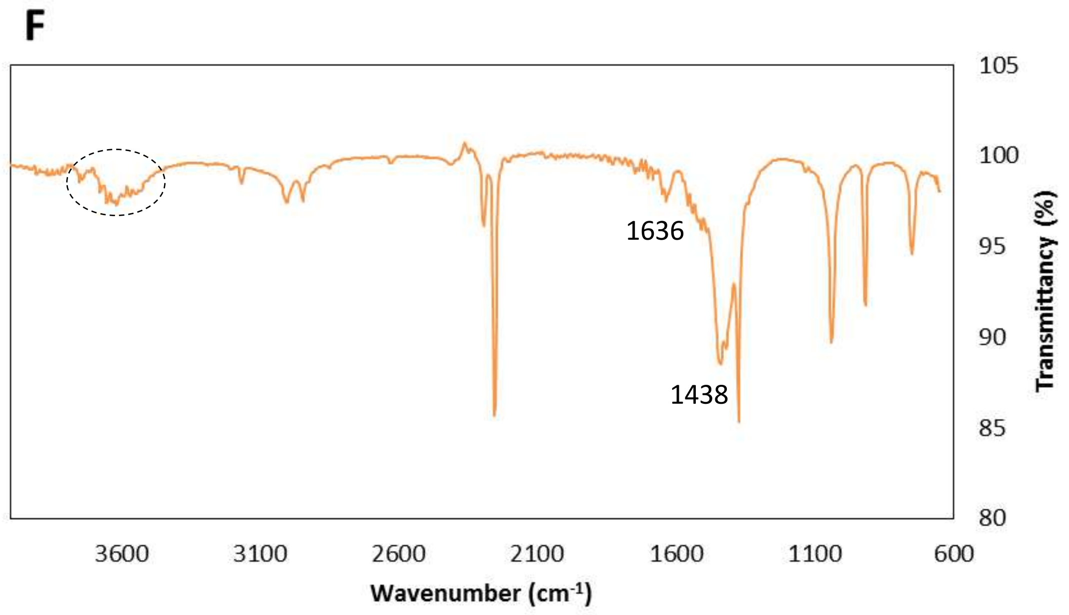
F
1636
1438
105
100
95
90
85
80
Transmittancy (%)
3600
3100
2600
2100
1600
1100
600
Wavenumber (cm-1)

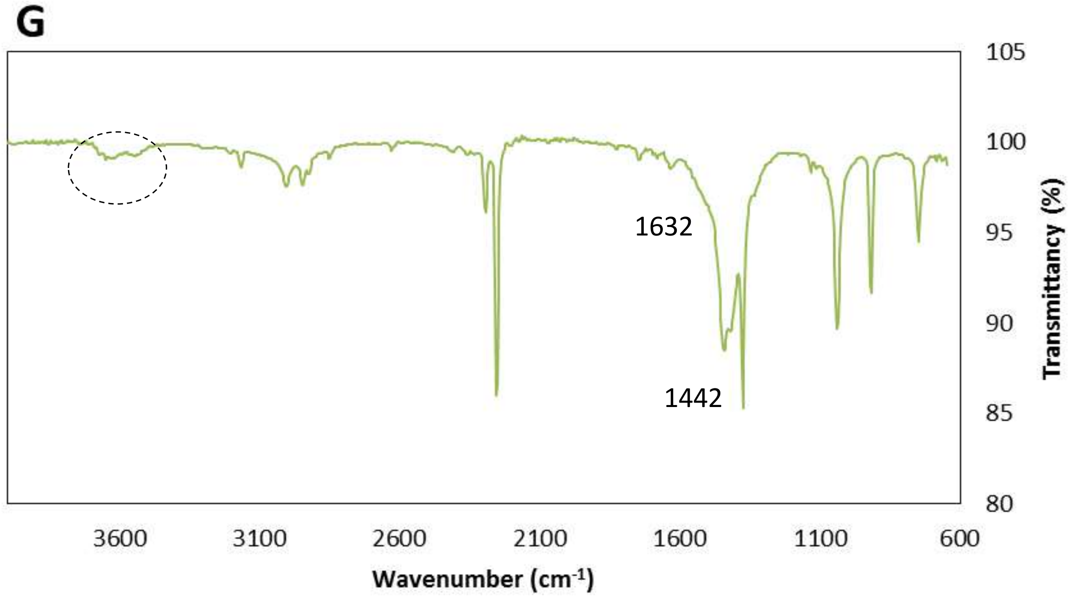

Fig. S7 FT-IR spectra of solid **Sulfo-Glu** and selected isotropic solutions/gels at the CGC of such gels. The measurements of the solutions were conducted at rt after all the **Sulfo-Glu** was dissolved and before the gel was formed. (A) solid **Sulfo-Glu**; (B) **Sulfo-Glu** dissolved in H_2O; (C) **Sulfo-Glu** gel in H_2O; (D) **Sulfo-Glu** dissolved in toluene; (E) **Sulfo-Glu** gel in toluene; (F) **Sulfo-Glu** dissolved in CH_3CN; (G) **Sulfo-Glu** gel in CH_3CN.

7. Variable temperature NMR experiments

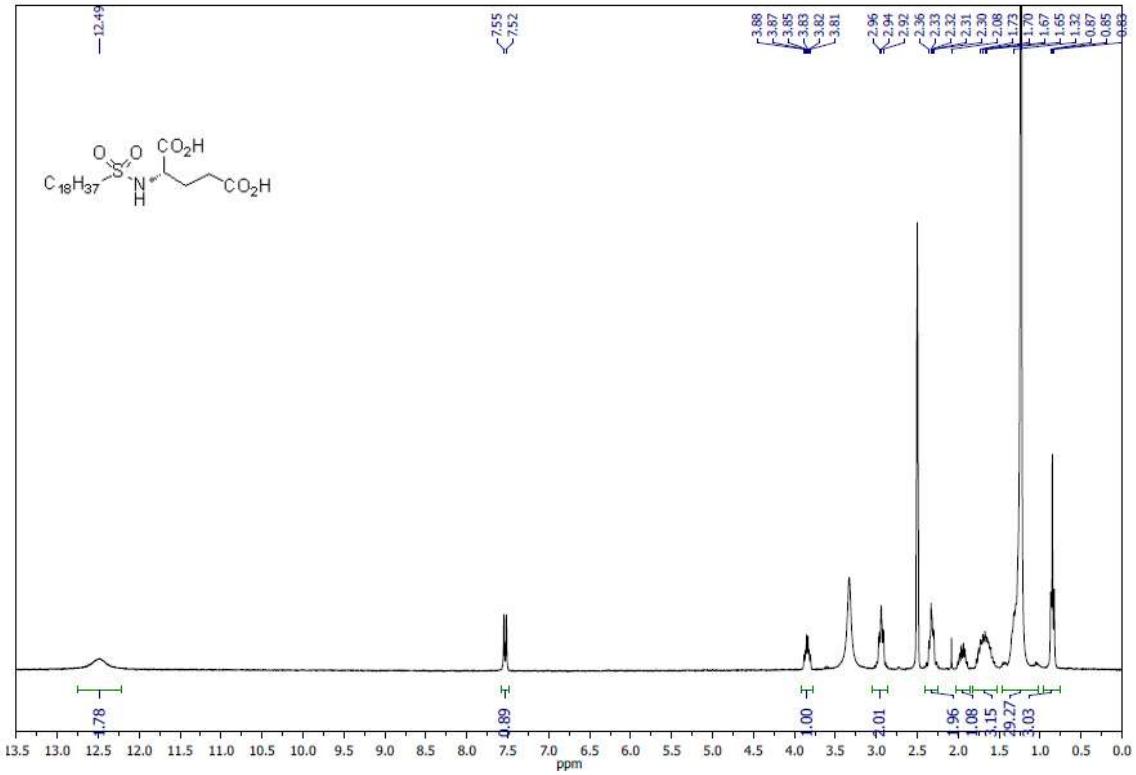

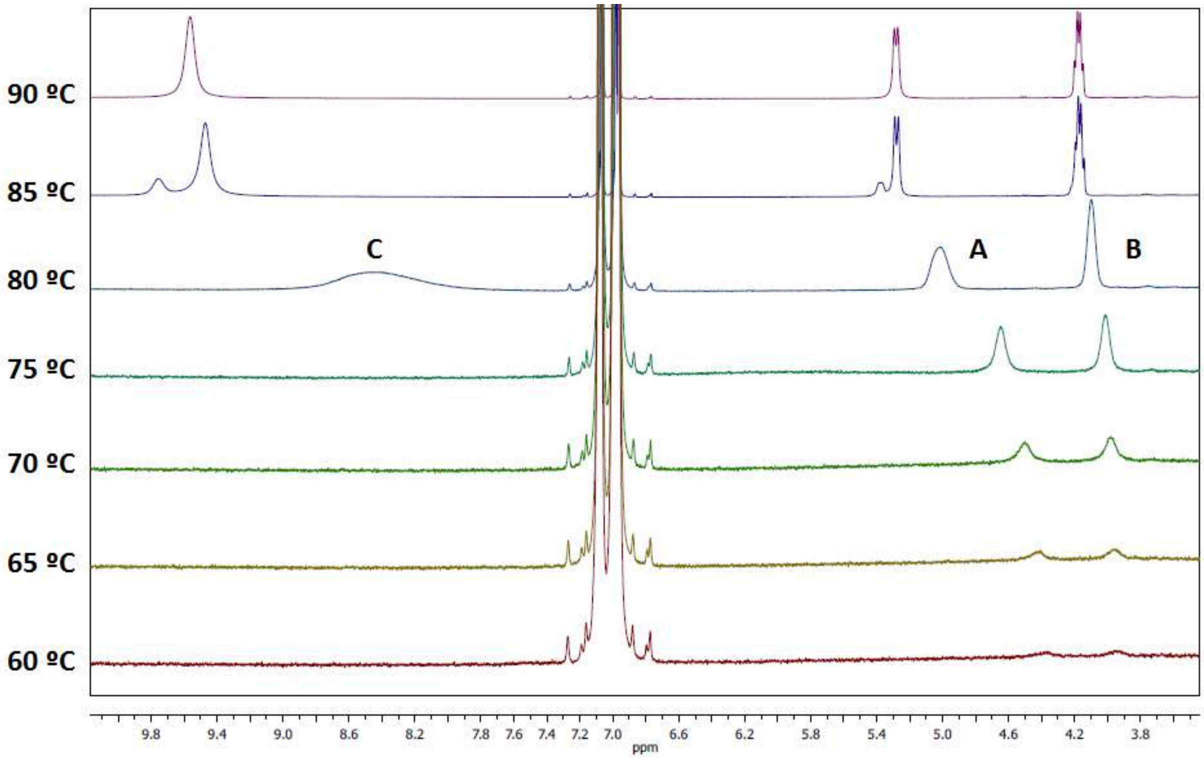

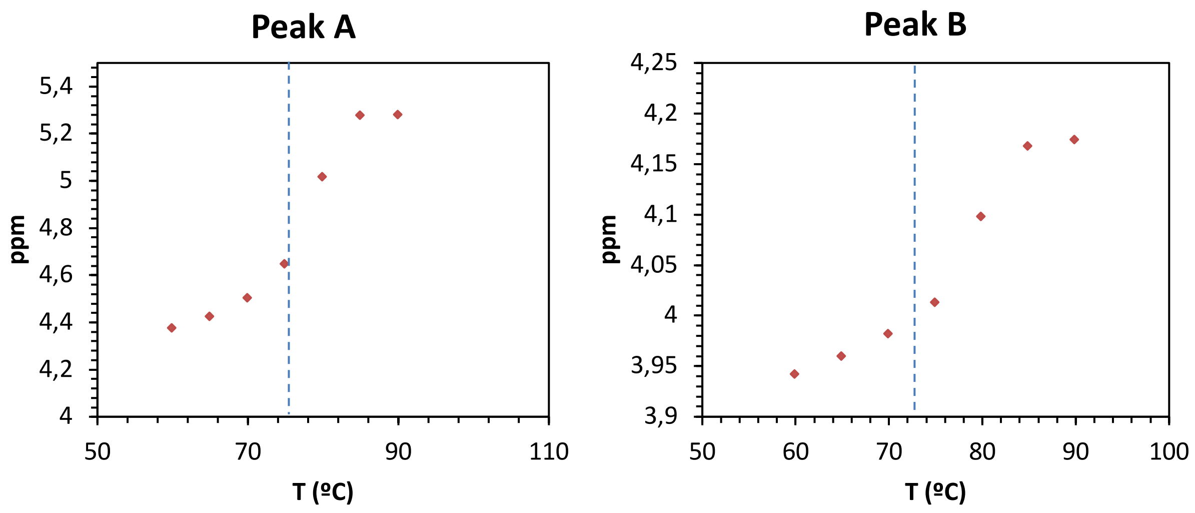

Fig. S8 1H-NMR spectrum of **Sulfo-Glu** in toluene (c = 30.2 g L^{-1}) and plot of chemical shifts of peaks **A** and **B** against temperature. The dotted line represents the middle of the curve.

8. Cartesian coordinates

Table S3 Cartesian coordinates for the structures of the lowest energy minima in chloroform.

C$_{18}$-Glu

6	14.341697	8.482035	-2.207525
6	13.472191	7.858921	-1.129595
6	14.267112	7.405022	0.085935
6	13.412004	6.778952	1.176195
6	14.206565	6.324097	2.390408
6	13.350287	5.697612	3.479786
6	14.143253	5.240184	4.694058
6	13.284641	4.614630	5.782090
6	14.074782	4.152633	6.996533
6	13.211039	3.532628	8.083665
6	13.995756	3.062438	9.298526
6	13.122405	2.456545	10.386092
6	13.898911	1.976602	11.602331
6	13.013350	1.395995	12.694331
6	13.785728	0.907938	13.909771
6	12.892770	0.365257	15.015186
6	13.686774	-0.105608	16.222386
6	12.834753	-0.650258	17.345420
7	13.366520	-0.520622	18.587698
6	12.674552	-0.995379	19.765895
6	13.644778	-0.862761	20.938002
6	13.109799	-1.208268	22.310596
6	14.165570	-0.952448	23.355646
8	13.817088	-1.148160	24.633615
8	11.748825	-1.210687	17.173403
6	12.258943	-2.445034	19.590769
8	12.963638	-3.324835	19.128877
8	11.048279	-2.672420	20.105607
8	15.302213	-0.583307	23.112048
8	16.003020	0.627887	19.627957
6	16.873086	-0.212153	19.814438
8	17.113059	-0.790374	20.983595
6	17.766670	-0.744831	18.711825
7	17.341766	-0.120862	17.475968
6	18.027290	-0.218105	16.306246
8	19.106830	-0.808112	16.221570
6	17.718263	-2.276813	18.637470
6	16.311937	-2.807738	18.430383
6	16.270121	-4.287201	18.141712
8	17.248269	-4.960389	17.881323
8	15.054279	-4.853823	18.159976
6	17.316263	0.389780	15.119009
6	18.232808	0.927313	14.034494
6	17.449192	1.411487	12.824392
6	18.318923	1.985757	11.718121
6	17.522919	2.446664	10.507028
6	18.381130	3.042876	9.402570
6	17.585048	3.495609	8.188637
6	18.442283	4.104167	7.089891
6	17.648596	4.554253	5.873377
6	18.506841	5.169099	4.778903
6	17.715664	5.619212	3.560676
6	18.576715	6.234775	2.468812
6	17.789835	6.685645	1.248009
6	18.656804	7.296894	0.158242
6	17.876878	7.749507	-1.066226
6	18.752192	8.356200	-2.153433

6	17.968377	8.804609	-3.374995
1	11.766745	-0.405633	19.948193
1	14.518051	-1.491437	20.724081
1	14.005512	0.169093	20.948944
1	12.219513	-0.616308	22.555483
1	12.801205	-2.258617	22.387703
1	14.221867	0.004415	18.729769
1	12.893666	-1.430705	24.707072
1	10.856981	-3.621815	20.028647
1	14.377408	-0.908137	15.921847
1	14.319544	0.708357	16.602234
1	12.285766	-0.466106	14.637435
1	12.180499	1.140581	15.327399
1	14.399230	1.732610	14.305312
1	14.502427	0.131641	13.599886
1	12.420348	0.566155	12.282253
1	12.281406	2.154442	13.009779
1	14.641466	1.225566	11.291080
1	14.486671	2.811787	12.014284
1	12.548544	1.616520	9.966560
1	12.371792	3.196377	10.703086
1	14.565829	3.907342	9.715534
1	14.754392	2.328937	8.984335
1	12.649895	2.684460	7.663064
1	12.449150	4.260320	8.402081
1	14.843183	3.429902	6.681080
1	14.633835	5.004307	7.414787
1	12.730645	3.761181	5.362654
1	12.516749	5.336188	6.100043
1	14.697542	6.095186	5.111942
1	14.915820	4.522062	4.378213
1	12.798923	4.841690	3.061924
1	12.580264	6.417151	3.797111
1	14.980326	5.607233	2.074581
1	14.759503	7.180576	2.807081
1	12.861534	5.921804	0.759679
1	12.641324	7.497947	1.493130
1	14.820044	8.262470	0.500906
1	15.041450	6.688764	-0.230254
1	12.924659	7.000344	-1.542966
1	12.702210	8.575358	-0.810573
1	15.103000	7.775672	-2.558364
1	13.757969	8.800207	-3.076591
1	14.874684	9.360940	-1.826209
1	18.799418	-0.448818	18.943130
1	18.360530	-2.590577	17.811908
1	18.149776	-2.688886	19.553535
1	15.666456	-2.602311	19.296159
1	15.827966	-2.299966	17.582919
1	16.541711	0.494393	17.536192
1	14.364594	-4.212897	18.428214
1	16.450162	-0.505427	21.657030
1	16.617107	1.168657	15.451328
1	16.685319	-0.408372	14.698800
1	18.841437	1.747777	14.438303
1	18.940339	0.143627	13.737821
1	16.850891	0.578340	12.423700
1	16.714822	2.168808	13.140338
1	18.904005	2.829216	12.114051
1	19.058192	1.233276	11.405594
1	16.768940	3.184130	10.823707
1	16.946297	1.598752	10.105514
1	18.949772	3.895381	9.803804

1	19.136384	2.306239	9.089380
1	17.021054	2.641497	7.782240
1	16.820498	4.223315	8.502231
1	19.001092	4.960493	7.496910
1	19.206102	3.375857	6.777730
1	16.878568	5.277054	6.184829
1	17.091195	3.697560	5.463234
1	19.060995	6.026602	5.189808
1	19.274561	4.444583	4.467411
1	17.159994	4.762266	3.148659
1	16.944322	6.341297	3.870490
1	19.129854	7.091994	2.881665
1	19.345258	5.510294	2.159237
1	17.019742	7.410425	1.554607
1	17.232981	5.829765	0.835378
1	19.211643	8.152481	0.572205
1	19.423770	6.569141	-0.147522
1	17.318249	6.895657	-1.480856
1	17.108572	8.478025	-0.764023
1	19.306563	9.207856	-1.734915
1	19.515833	7.623733	-2.450463
1	17.221901	9.562018	-3.110492
1	18.618105	9.233796	-4.143444
1	17.428628	7.965535	-3.828723

Click-Glu

7	0.470235	2.957404	0.191852
7	-0.652258	2.687812	0.890678
6	-1.318100	3.823939	1.201322
6	-0.542278	4.835163	0.670483
7	0.535600	4.258479	0.060503
6	-1.016560	1.320837	1.201305
6	-0.963087	1.117881	2.719403
8	-1.441937	-0.056056	3.169823
6	-0.714053	6.316123	0.736483
6	-0.148406	7.058507	-0.467740
6	-0.272014	8.568916	-0.350876
6	0.277328	9.303685	-1.563540
6	0.161553	10.816996	-1.473073
6	0.724961	11.537184	-2.688118
6	0.602830	13.051059	-2.616436
6	1.180909	13.763913	-3.828868
6	1.055280	15.278017	-3.766837
6	1.641876	15.986843	-4.977523
6	1.515956	17.501181	-4.920854
6	2.109867	18.206600	-6.130022
6	1.982845	19.721063	-6.079065
6	2.589804	20.424091	-7.283114
6	2.442013	21.938008	-7.246066
6	3.063916	22.629083	-8.447301
6	-2.346006	0.941650	0.540583
6	-2.308686	1.200934	-0.967183
6	-3.505556	0.580660	-1.656956
8	-3.495917	-0.563838	-2.070846
8	-4.601216	1.334874	-1.768752
8	-0.491848	1.920726	3.485486
7	-4.398749	3.697465	-0.353443
6	-4.069673	5.015935	-0.444236
6	-4.318918	5.582619	0.788903
7	-4.769645	4.559409	1.560157

7	-4.825617	3.418772	0.855496
6	-3.509089	5.624283	-1.680734
6	-3.572712	7.145085	-1.684468
6	-3.017449	7.776387	-2.951038
6	-3.092855	9.295150	-2.928800
6	-2.520691	9.963701	-4.168783
6	-2.616535	11.480961	-4.127027
6	-2.032670	12.170594	-5.350003
6	-2.145206	13.686380	-5.299610
6	-1.556192	14.387334	-6.513758
6	-1.678924	15.902112	-6.458469
6	-1.089478	16.610073	-7.668371
6	-1.220937	18.124009	-7.610090
6	-0.631143	18.837680	-8.816455
6	-0.777658	20.350272	-8.760526
6	-0.167836	21.068898	-9.955066
6	-0.334295	22.577507	-9.893683
6	-5.206900	4.550718	2.957389
6	-4.672257	3.290441	3.625194
8	-3.576460	2.815745	3.408403
6	-4.713447	5.790620	3.709765
6	-3.199327	5.873355	3.869280
6	-2.683644	5.080493	5.052372
8	-1.372606	4.786561	5.056430
8	-3.377161	4.755791	5.991894
8	-5.539039	2.834459	4.528738
1	-0.212145	0.709957	0.769469
1	-2.541644	-0.124727	0.705360
1	-3.168635	1.493941	1.013232
1	-2.259546	2.279741	-1.156543
1	-1.411635	0.744799	-1.395692
1	-4.478765	2.230480	-1.350093
1	-1.781590	-0.606547	2.449793
1	-1.782090	6.550463	0.853230
1	-0.230976	6.696298	1.649785
1	0.904187	6.781341	-0.598290
1	-0.656719	6.713414	-1.380482
1	-1.328422	8.847359	-0.202829
1	0.251432	8.910700	0.554341
1	-0.244914	8.952401	-2.467654
1	1.331589	9.026255	-1.708193
1	-0.896280	11.096829	-1.340799
1	0.674811	11.170898	-0.566524
1	1.782400	11.262130	-2.815799
1	0.215513	11.173361	-3.594730
1	-0.457969	13.327236	-2.502149
1	1.100815	13.416014	-1.705615
1	0.683528	13.394207	-4.739798
1	2.240483	13.490051	-3.941464
1	-0.007060	15.552194	-3.662785
1	1.544538	15.648664	-2.853548
1	2.702248	15.712658	-5.082044
1	1.150650	15.614562	-5.890734
1	0.453030	17.775574	-4.823889
1	1.999721	17.874479	-4.005716
1	1.624104	17.831645	-7.045088
1	3.170908	17.932214	-6.227244
1	0.918612	19.994655	-5.995259
1	2.455560	20.097374	-5.159400
1	3.655754	20.162349	-7.360240
1	2.125138	20.036527	-8.203968
1	1.373209	22.191652	-7.181527
1	2.891788	22.323107	-6.320338

1	2.622273	22.268124	-9.383630
1	2.922517	23.713606	-8.415605
1	4.140947	22.435969	-8.500920
1	-2.238833	3.788645	1.770799
1	-6.300303	4.531376	2.973512
1	-5.100053	6.678130	3.202131
1	-5.175429	5.776125	4.700569
1	-2.676718	5.569664	2.953855
1	-2.902824	6.915062	4.041035
1	-0.952713	5.017710	4.214985
1	-5.115808	2.107432	5.014900
1	-2.464314	5.293354	-1.793716
1	-4.039710	5.216201	-2.549579
1	-4.614313	7.465254	-1.540884
1	-3.018209	7.537732	-0.817879
1	-1.971699	7.461510	-3.092079
1	-3.563083	7.390941	-3.824174
1	-2.562701	9.670522	-2.038789
1	-4.140551	9.604591	-2.798936
1	-1.466964	9.666581	-4.292097
1	-3.039341	9.586841	-5.062656
1	-3.670262	11.775620	-4.011090
1	-2.106741	11.851752	-3.223260
1	-0.973724	11.886189	-5.459270
1	-2.532242	11.795896	-6.255854
1	-1.648382	14.057670	-4.388916
1	-3.203051	13.969611	-5.192906
1	-0.494465	14.110465	-6.615766
1	-2.045630	14.013544	-7.425597
1	-2.738992	16.178278	-6.355309
1	-1.188157	16.273793	-5.544700
1	-0.025849	16.339385	-7.767291
1	-1.573475	16.235898	-8.582988
1	-0.735007	18.496489	-6.694060
1	-2.282890	18.393908	-7.509709
1	0.435806	18.577568	-8.907911
1	-1.105078	18.458207	-9.734166
1	-1.843656	20.611511	-8.681901
1	-0.314116	20.727393	-7.834753
1	0.900573	20.813541	-10.019184
1	-0.618512	20.681454	-10.879355
1	0.118486	22.988478	-8.983665
1	0.134356	23.077323	-10.746751
1	-1.393121	22.858586	-9.884710
1	-4.218821	6.593667	1.152260

Sulfo-Glu

6	13.300686	-1.288916	-10.571068
6	12.663631	-2.293392	-9.628335
6	11.347435	-1.803588	-9.045260
6	10.633822	-2.833121	-8.184363
6	9.342863	-2.307196	-7.578679
6	8.541328	-3.345486	-6.810592
6	7.265582	-2.773804	-6.214663
6	6.369764	-3.790602	-5.525388
6	5.086511	-3.162519	-5.006759
6	4.108441	-4.120979	-4.344921
6	2.822275	-3.416060	-3.946168
6	1.775151	-4.275427	-3.253824
6	0.498050	-3.484758	-3.019807

6	-0.609390	-4.191009	-2.251994
6	-1.862222	-3.327939	-2.238592
6	-3.016471	-3.819298	-1.374762
6	-4.178125	-2.857827	-1.535718
16	-5.671027	-3.211310	-0.617213
7	-5.102590	-3.211515	0.979067
6	-5.940602	-3.922636	1.960010
6	-7.379219	-3.407296	2.062909
6	-8.037742	-3.731203	3.397369
6	-9.395514	-3.131777	3.617235
8	-10.018188	-2.811202	2.465038
8	-6.590180	-2.095773	-0.865295
8	-6.116100	-4.582829	-0.870188
6	-5.865607	-5.432329	1.781947
8	-6.829237	-6.151392	2.026716
8	-4.671084	-5.880904	1.462704
8	-9.905815	-2.959383	4.707594
8	-6.915950	-8.836909	2.007352
6	-5.754712	-9.347263	1.656702
8	-4.720296	-8.674824	1.550610
6	-5.792431	-10.827632	1.410070
6	-5.897493	-11.227969	-0.066849
6	-4.658920	-10.937157	-0.920379
7	-4.371077	-9.530105	-1.226739
16	-5.602714	-8.674558	-1.992315
8	-6.454712	-7.995133	-1.009203
6	-4.651548	-11.794803	-2.179059
8	-5.344738	-12.778189	-2.335738
8	-3.713298	-11.404801	-3.057173
6	-4.689464	-7.422938	-2.910796
6	-3.432909	-7.954900	-3.573051
6	-2.704711	-6.928952	-4.434677
6	-1.369866	-7.491341	-4.905366
6	-0.488584	-6.544774	-5.705872
6	0.813194	-7.214137	-6.125197
6	1.814833	-6.305090	-6.820538
6	3.088172	-7.037464	-7.218702
6	4.148854	-6.157608	-7.862249
6	5.399058	-6.927419	-8.262423
6	6.464186	-6.082272	-8.945867
6	7.697456	-6.881865	-9.340999
6	8.739981	-6.088046	-10.116005
6	9.973718	-6.907050	-10.469216
6	10.985430	-6.173872	-11.339218
6	12.244853	-6.985580	-11.612062
6	13.233729	-6.283725	-12.528390
8	-6.202007	-9.630467	-2.932091
1	-5.429248	-3.741366	2.917368
1	-7.349977	-2.320972	1.927240
1	-7.973640	-3.805973	1.234835
1	-8.160033	-4.814621	3.504264
1	-7.412821	-3.415359	4.239498
1	-4.966604	-2.246839	1.273351
1	-10.892519	-2.461611	2.698150
1	-4.702699	-6.878061	1.464245
1	-3.901758	-1.835175	-1.255944
1	-4.532780	-2.817489	-2.572493
1	-2.720640	-3.883772	-0.321601
1	-3.318972	-4.831789	-1.673292
1	-2.216928	-3.213634	-3.274677
1	-1.587767	-2.314209	-1.912114
1	-0.279603	-4.407369	-1.225921
1	-0.829958	-5.168160	-2.708926

1	0.746244	-2.550489	-2.494021
1	0.104097	-3.167545	-3.998581
1	2.169696	-4.657600	-2.300941
1	1.553336	-5.165193	-3.864513
1	2.375715	-2.970961	-4.849760
1	3.064893	-2.562050	-3.295718
1	4.574374	-4.587569	-3.464557
1	3.876051	-4.948413	-5.034812
1	5.335844	-2.356612	-4.299989
1	4.578217	-2.662364	-5.846728
1	6.912344	-4.272597	-4.698647
1	6.124719	-4.599314	-6.233409
1	6.689788	-2.279203	-7.013635
1	7.523281	-1.973894	-5.504435
1	9.162562	-3.781794	-6.014583
1	8.290595	-4.182697	-7.482505
1	9.571187	-1.458984	-6.916048
1	8.711337	-1.890480	-8.379841
1	11.306148	-3.177442	-7.384588
1	10.417423	-3.726515	-8.791471
1	10.680128	-1.496528	-9.866301
1	11.523468	-0.891064	-8.455950
1	13.357995	-2.530691	-8.810746
1	12.491770	-3.244120	-10.153823
1	13.518046	-0.345889	-10.057270
1	14.239115	-1.658587	-10.995597
1	12.629665	-1.054154	-11.406000
1	-3.766735	-11.262423	-0.364050
1	-6.067678	-12.306307	-0.103975
1	-6.778816	-10.764071	-0.523676
1	-6.647912	-11.242987	1.945935
1	-4.880961	-11.257360	1.838991
1	-4.101329	-9.035104	-0.377550
1	-6.830799	-7.846232	2.098685
1	-3.754175	-12.024350	-3.802444
1	-4.489805	-6.622722	-2.189608
1	-5.428903	-7.044043	-3.624466
1	-2.751481	-8.312247	-2.792891
1	-3.679637	-8.838073	-4.174688
1	-3.322184	-6.636864	-5.295147
1	-2.537014	-6.006172	-3.857872
1	-0.803349	-7.828089	-4.022966
1	-1.553501	-8.401897	-5.494771
1	-0.267598	-5.650811	-5.102048
1	-1.026049	-6.180754	-6.593570
1	1.292644	-7.648508	-5.233561
1	0.583807	-8.071274	-6.776022
1	1.354599	-5.851090	-7.710594
1	2.067753	-5.465241	-6.154133
1	3.516425	-7.525272	-6.328517
1	2.832443	-7.858889	-7.904985
1	4.423777	-5.346919	-7.168485
1	3.725598	-5.659682	-8.747591
1	5.830705	-7.408928	-7.370493
1	5.114068	-7.754214	-8.930609
1	6.032602	-5.611291	-9.841885
1	6.758805	-5.249543	-8.285872
1	8.158518	-7.310449	-8.436806
1	7.385976	-7.746332	-9.946833
1	9.040218	-5.201049	-9.535024
1	8.284859	-5.696272	-11.037996
1	10.463821	-7.242178	-9.541325
1	9.660403	-7.828846	-10.982391

1	10.512221	-5.898323	-12.293571
1	11.261600	-5.219468	-10.862746
1	12.728269	-7.225024	-10.653358
1	11.959316	-7.952934	-12.048835
1	13.558172	-5.324568	-12.109517
1	14.131263	-6.885547	-12.699341
1	12.785720	-6.072506	-13.505642

9. NMR spectra

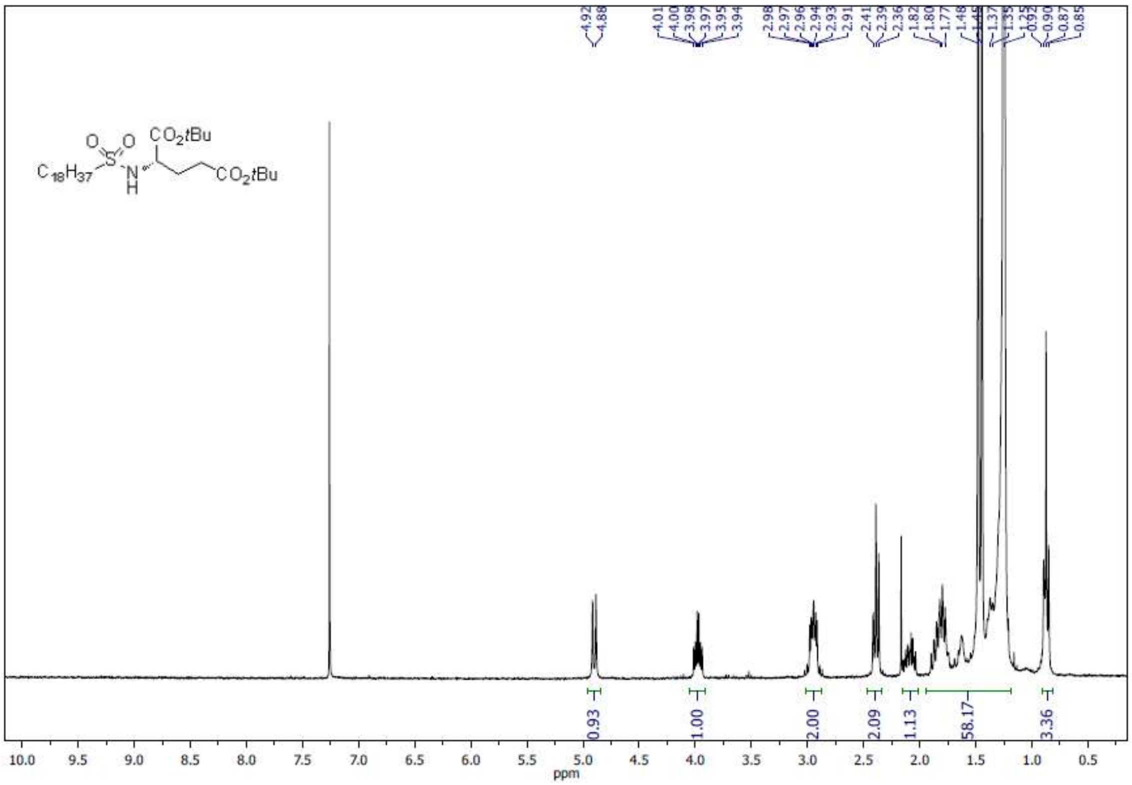

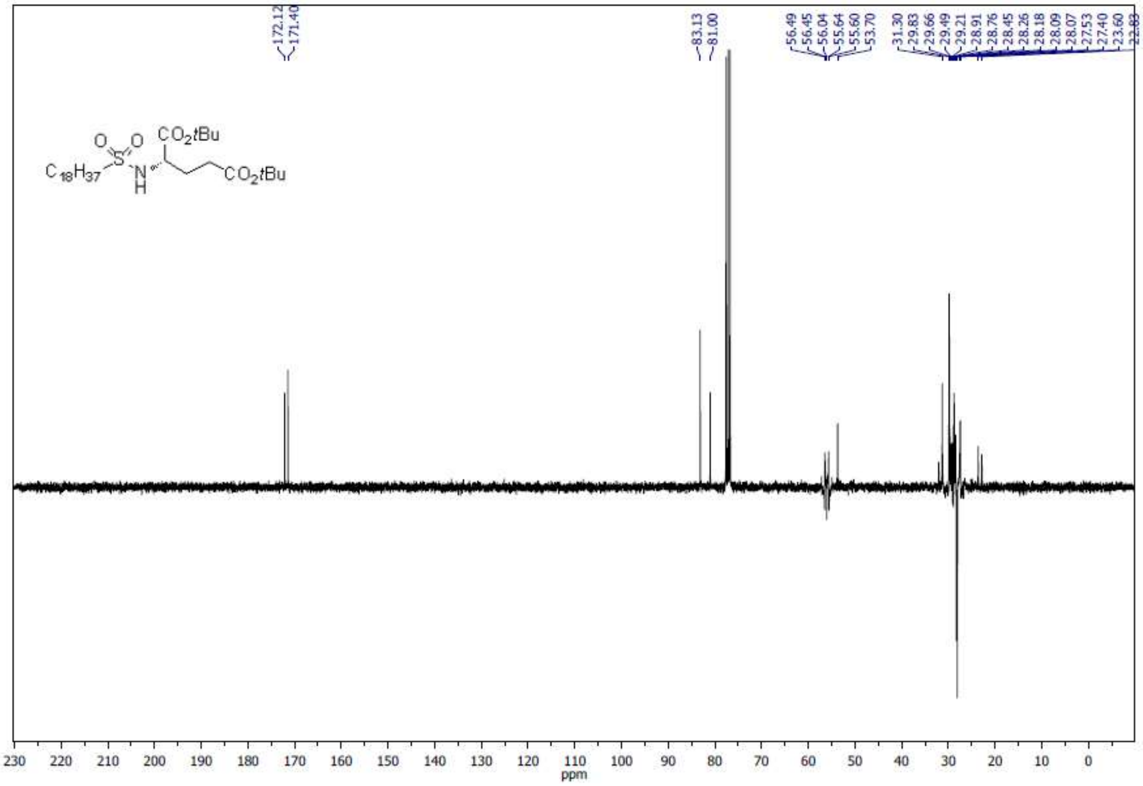

Fig. S9 ^{1}H NMR and ^{13}C-NMR-APT spectra of **Sulfo-Glu ester**.

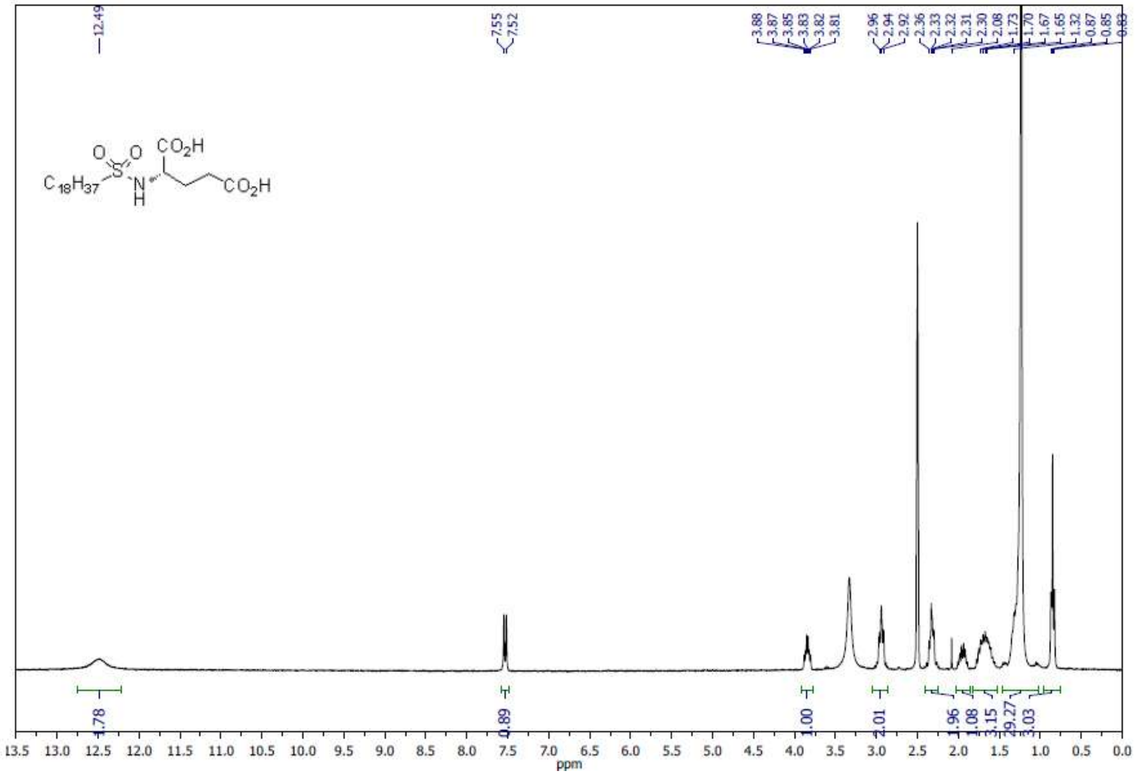

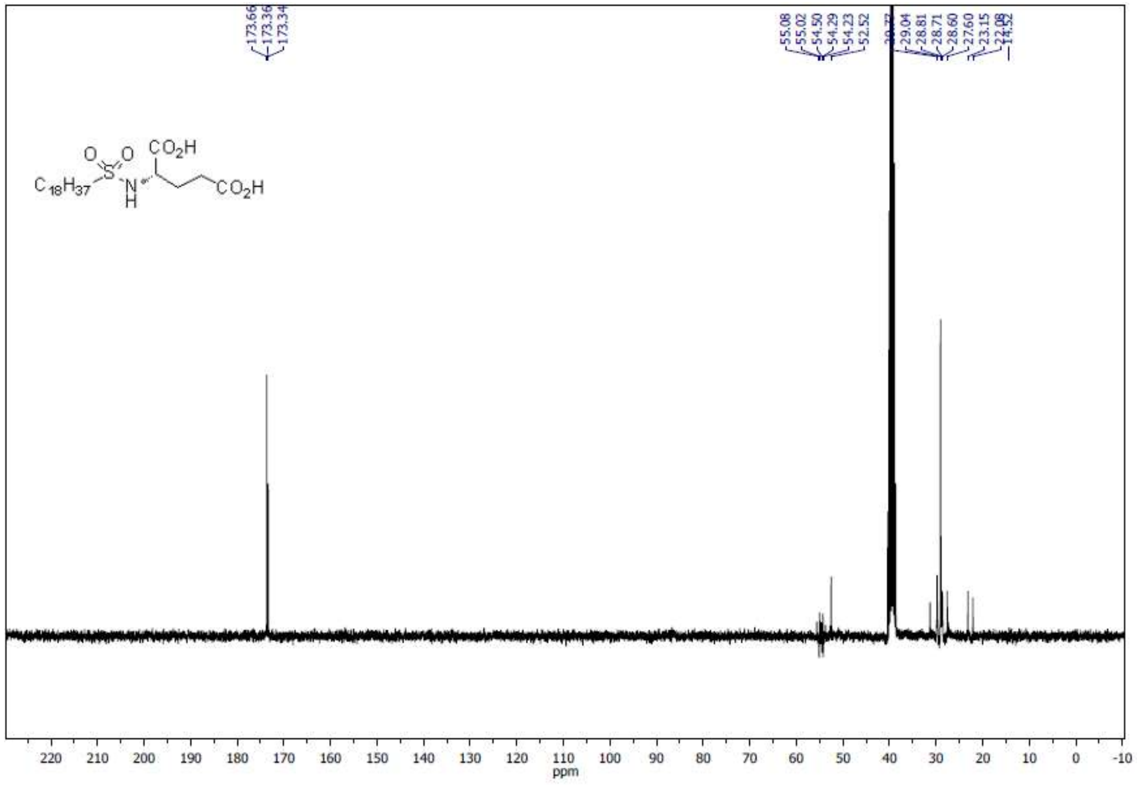

Fig. S10 ^{1}H NMR and ^{13}C-NMR-APT spectra of **Sulfo-Glu**.
